# Complex Interactions Between Membrane-Bound Organelles, Biomolecular Condensates and the Cytoskeleton

**DOI:** 10.3389/fcell.2020.618733

**Published:** 2020-12-21

**Authors:** Max Koppers, Nazmiye Özkan, Ginny G. Farías

**Affiliations:** Cell Biology, Neurobiology and Biophysics, Department of Biology, Faculty of Science, Utrecht University, Utrecht, Netherlands

**Keywords:** cytoskeleton, organelle contacts, organelle dynamics, ER, neurons, membraneless organelles, biomolecular condensates, membrane-bound organelles

## Abstract

Membrane-bound and membraneless organelles/biomolecular condensates ensure compartmentalization into functionally distinct units enabling proper organization of cellular processes. Membrane-bound organelles form dynamic contacts with each other to enable the exchange of molecules and to regulate organelle division and positioning in coordination with the cytoskeleton. Crosstalk between the cytoskeleton and dynamic membrane-bound organelles has more recently also been found to regulate cytoskeletal organization. Interestingly, recent work has revealed that, in addition, the cytoskeleton and membrane-bound organelles interact with cytoplasmic biomolecular condensates. The extent and relevance of these complex interactions are just beginning to emerge but may be important for cytoskeletal organization and organelle transport and remodeling. In this review, we highlight these emerging functions and emphasize the complex interplay of the cytoskeleton with these organelles. The crosstalk between membrane-bound organelles, biomolecular condensates and the cytoskeleton in highly polarized cells such as neurons could play essential roles in neuronal development, function and maintenance.

## Introduction

Cells execute numerous biochemical processes that need to be spatiotemporally regulated in the crowded cellular environment. This organization can be achieved by compartmentalization of the cell into functionally and morphologically distinct domains that include both membrane-bound organelles, such as the endoplasmic reticulum (ER), mitochondria and the endo-lysosomal system, and less well-characterized compartments that lack a lipid membrane called membraneless organelles or biomolecular condensates. These biomolecular condensates form by a process called phase separation that drives liquid-liquid demixing from the surrounding environment to create a local concentration of specific proteins and RNAs thereby promoting or inhibiting certain biochemical reactions (Banani et al., 2017; Boeynaems et al., 2018). Phase separation is driven by multivalent protein-protein, RNA-RNA and protein-RNA interactions (Boeynaems et al., 2018; Tauber et al., 2020). Biomolecular condensates have been identified in both the nucleus (e.g., the nucleolus, nuclear speckles, PML bodies and Cajal bodies) and the cytoplasm [e.g., germ granules, processing bodies (P-bodies), stress granules (SGs), and RNP transport granules] and a quickly increasing amount of work is revealing that these condensates are involved in many different cellular processes such as cell division, ribosome biogenesis, regulation of RNA metabolism, and signal transduction (Banani et al., 2017; Boeynaems et al., 2018; Sabari et al., 2020).

Another essential component important for cellular organization is the cytoskeleton. The cytoskeleton consists of actin filaments, intermediate filaments, and microtubules (MTs) that form a dynamic and highly extensive network throughout the cell. The cytoskeleton determines cell shape, cell polarity, and mechanics and regulates cell division. In addition, it provides the tracks along which proteins, mRNAs and organelles can be transported driven by motor proteins, which is most prominent in highly polarized and morphologically complex cells such as neurons. This intracellular transport ensures the proper distribution, organization and dynamics of both membrane-bound organelles and biomolecular condensates. In animal cells, long-range organelle transport is mainly achieved on the microtubule cytoskeleton whereas the actin cytoskeleton regulates short-range cargo transport (Hirokawa et al., 2009; Burute and Kapitein, 2019). In plant cells, these roles are reversed with the actin cytoskeleton mainly driving the rapid motion of organelles called cytoplasmic streaming (Geitmann and Nebenfuhr, 2015).

The cytoskeleton, membrane-bound organelles and biomolecular condensates function together and are known to interact and communicate with each other. Contacts between membrane-bound organelles have been observed for many years. For instance, the ER can make extensive and dynamic contacts with other membrane-bound organelles such as mitochondria, the trans-Golgi network (TGN), the endo-lysosomal system, the plasma membrane and lipid droplets at so-called membrane contact sites (MCSs) (reviewed in Phillips and Voeltz, 2016; Fowler et al., 2019). MCSs, mediated by tethering proteins, provide an alternative to vesicle-dependent inter-organelle communication by allowing the exchange of small molecules and ions between organelles that is essential to maintain cellular homeostasis (Scorrano et al., 2019). More recent evidence has shown that these interactions play important roles in organelle positioning, dynamics and function. In addition to contacts between membrane-bound organelles, interactions between membrane-bound organelles and biomolecular condensates have emerged increasingly over the past few years (Zhao and Zhang, 2020). These discoveries have brought exciting new prospects to inter-organelle communication within the cell, but it remains largely unclear how these contacts are formed and regulated and what roles they play in cellular function. In addition, the role of the cytoskeleton in these contacts and the interplay of these organelles and condensates with the cytoskeleton are just beginning to emerge. Understanding the complex relationship and interplay between the cytoskeleton, membrane-bound organelles and biomolecular condensates is imperative since dysfunction of each of these components and the dysregulation of their interactions are known to be involved in several neurodegenerative diseases including hereditary spastic paraplegia (HSP) and amyotrophic lateral sclerosis (ALS) (Fowler et al., 2019; Sleigh et al., 2019; Zbinden et al., 2020).

In this review, we will discuss the current knowledge on the intricate interplay between the cytoskeleton, membrane-bound organelles and biomolecular condensates and expand the idea that this interplay is essential for many crucial cellular processes. In the first section, we will discuss how the cytoskeleton affects the organization and dynamics of membrane-bound organelles such as the ER and mitochondria; and conversely how dynamic membrane-bound organelles can affect cytoskeletal organization. Then we will explore complex interactions between membrane-bound organelles in conjunction with the cytoskeleton. In the second section, we will explore the role of the cytoskeleton in the transport and dynamics of biomolecular condensates and discuss how these condensates can influence the cytoskeleton. In the third section, we will discuss the interactions between membrane-bound organelles and biomolecular condensates and emphasize the intricate interplay of these contacts with the cytoskeleton. Finally, we will explore the link of these complex interactions with neurodegenerative diseases and point out open questions in this field.

## The Interplay Between Membrane-Bound Organelles and the Cytoskeleton

The correct positioning of organelles, mediated by motor protein-driven intracellular transport along the microtubule and actin cytoskeleton, is essential for many cellular functions (reviewed in Rogers and Gelfand, 2000; Bonifacino and Neefjes, 2017; Burute and Kapitein, 2019). However, it has only recently become increasingly recognized that there is an intricate interplay between various membrane-bound organelles and the cytoskeleton that extends beyond single organelle movement. For instance, membrane-bound organelles are remodeled by the cytoskeleton and reciprocally, dynamic membrane-bound organelles contribute to cytoskeletal organization. Moreover, complex inter-organelle interactions have been found to play important roles in the transport, organization and dynamics of membrane-bound organelles in coordination with the cytoskeleton. In this section, we discuss this reciprocal crosstalk and their important cellular functions which are essential to maintain cellular homeostasis.

### The Role of the Cytoskeleton in Membrane-Bound Organelle Organization

#### The Influence of the Cytoskeleton on the ER Network

The ER is the largest membrane-bound organelle and is involved in many crucial cellular functions including protein synthesis and processing, calcium storage and lipid metabolism (Rapoport, 2007; Fagone and Jackowski, 2009; Braakman and Hebert, 2013; Schwarz and Blower, 2016). The ER consists of dynamic tubules and sheets, which form a continuous interconnected network throughout the cell (English et al., 2009; Friedman and Voeltz, 2011; Goyal and Blackstone, 2013). In unpolarized cells, ER sheets are localized to the perinuclear area whereas the interconnected tubules can extend throughout the periphery of the cell (Shibata et al., 2006; Chen et al., 2013). In polarized neurons, it has been shown that ER tubules can localize to both structurally and functionally different neuronal compartments, the somatodendritic and axonal domains; however, ER sheets are excluded from the axon (Wu et al., 2017; Farias et al., 2019).

The morphology of the ER is maintained by ER-shaping proteins. For instance, ER-resident proteins such as CLIMP63 generate the flattened structure of the sheets, Reticulons induce the high curvature of ER tubules, and the GTPase Atlastin-1 induces homo-fusion of tubules to generate a reticular network. The relative abundance of specific ER-shaping proteins regulates the sheet-to-tubule ratio and fusion between tubules, thus controlling the ER network (reviewed in Zhang and Hu, 2016; Wang and Rapoport, 2019). In addition, other factors may cooperate with these ER-shaping proteins to rearrange the ER network, as ER remodeling occurs in a timescale of milliseconds (Nixon-Abell et al., 2016; Guo et al., 2018).

Although a reticular ER network can be formed *in vitro* by ER-shaping proteins in absence of the cytoskeleton (Dreier and Rapoport, 2000), the ER can rearrange its network in response to cellular demands and this relies on its interaction with the cytoskeleton. First evidence for a role of MTs in ER organization came from experiments performed with the MT-depolymerizing drug nocodazole, which resulted in the retraction and interconversion of ER tubules into ER sheets (Terasaki et al., 1986). A similar phenotype has been observed in neurons, in which nocodazole induced the retraction of ER tubules from dendrites and the axon into the soma and their interconversion to somatic ER sheets (Farias et al., 2019).

**FIGURE 1:**
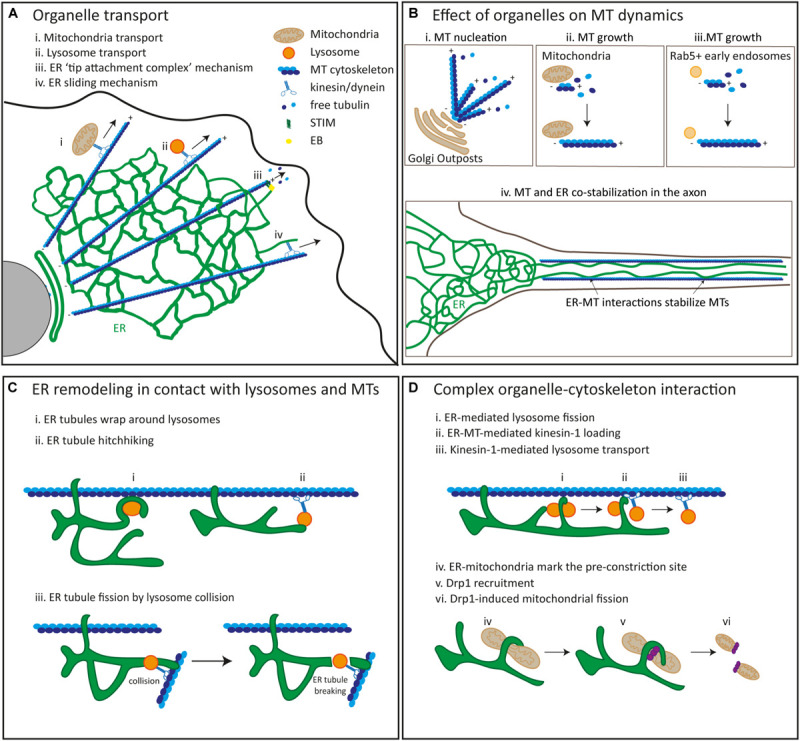
The interplay and interactions between membrane-bound organelles and the cytoskeleton. **(A)** MT-dependent transport of membrane-bound organelles such as mitochondria (i), lysosomes (ii) and the ER (iii) and (iv) in mammalian cells. ER tubules and microtubules can elongate together at the tip of a growing MT (TAC; iii) or ER tubules can be pulled out by microtubules via motor-dependent sliding (iv). **(B)** MT dynamics is mediated by membrane-bound organelles. MT nucleation can be induced by Golgi outposts (i). MT growth can be regulated by mitochondria (ii) and Rab5 + early endosomes (iii). MTs and the ER colocalize and co-stabilize each other in axons via an interplay between MTs and the ER (iv). **(C)** ER remodeling is regulated by interactions between the ER and lysosomes in conjunction with the cytoskeleton. ER tubules wrap around lysosomes (i). ER tubules are pulled out by hitchhiking on moving lysosomes (ii). Fission of ER tubules is induced by lysosome collisions (iii). **(D)** Lysosome fission is regulated by an interaction between the ER and lysosomes in association with the cytoskeleton (i, ii, iii). ER tubules induce lysosome fission (i), ER-MT interactions promote kinesin-1 loading onto lysosomes (ii) and lysosome transport is driven by kinesin-1 along MTs. Mitochondrial fission is mediated by ER tubules (iv, v, vi). ER-mitochondria contacts mark the pre-constriction site (iv), Drp1 is recruited to this pre-constriction site (v) and promotes mitochondrial fission (vi).

ER network rearrangements can be mediated by the cytoskeleton via four different mechanisms. First, the ER can form contacts with polymerizing MTs at their growing plus ends through the interaction between the ER protein STIM1 and the MT-associated protein EB1 (Figure 1A). Through this “tip attachment complex” (TAC) growing ER tubules can be pulled out by the growing plus end of dynamic MTs (Waterman-Storer and Salmon, 1998; Grigoriev et al., 2008; Rodriguez-Garcia et al., 2020). This TAC mechanism is also involved the reshaping and positioning of the ER important for dendritic spine morphology in hippocampal neurons and for axonal growth cone dynamics in DRG sensory neurons (Pchitskaya et al., 2017; Pavez et al., 2019). A second mechanism that involves an interaction between the ER and MT cytoskeleton is the “sliding mechanism,” in which newly produced ER tubules are coupled to MT-bound motor proteins such as kinesin-1 and dynein and are thereby extended along stable MTs (Figure 1A; Wozniak et al., 2009; Friedman et al., 2010). Knockdown of kinesin-1 and dynein was shown to impair anterograde and retrograde movement of ER tubules along the axon in rat hippocampal neurons, while knockdown of EBs 1–3 did not affect axonal ER distribution but does impair dendritic movement of the ER (Farias et al., 2019). In addition, a newly developed imaging technology called GI-SIM has revealed that newly extended ER tubules via TAC and sliding mechanisms meet and fuse with pre-existing tubules. This suggests a possible role of TAC and sliding mechanisms in maintaining a dynamic reticular ER network (Guo et al., 2018).

A third and recently identified mechanism for ER tubule rearrangement is a depolymerizing TAC (dTAC) mechanism in which newly formed ER tubules can be attached to the depolymerizing end of MTs and can be pulled out while MTs retract. It was suggested that molecules other than STIM1 and EB might play a role in this dTAC mechanism (Guo et al., 2018). However, recent evidence from *in vitro* assays revealed that at least the same EB protein might be involved in both TAC and dTAC mechanisms in which the amount of the forces applied are different (Rodriguez-Garcia et al., 2020).

Guo et al., also identified another way of MT-dependent ER network rearrangement, the so-called “hitchhiking” mechanism, in which an organelle takes advantage of the transport machinery of another organelle for their translocation. This will be further discussed in section “Complex Inter-Organelle Interactions in Coordination With the Cytoskeleton.”

GI-SIM also revealed a remodeling mechanism in which ER tubules can be extended without any obvious direct or indirect MT involvement called *de novo* budding. These events were rare and it remains unknown whether the actin cytoskeleton, intermediate filaments or another unseen mediator could be involved in this *de novo* budding of the ER.

In addition to the involvement of MTs in ER rearrangement, the actin cytoskeleton might also contribute to the remodeling of the ER network. Actin filaments were observed to localize to polygons occupied by surrounding ER. Depolymerization of actin filaments with latrunculin treatment led to a fluctuation in the sheet-to-tubule ratio by increasing the transformation of sheets to tubules and it led to a change in size and morphology of ER sheets in mammalian cells (Joensuu et al., 2014). This suggests an interaction between actin and the ER that supports ER sheet formation.

The actin-based motor protein myosin Va has been shown to be involved in ER motility in animal cells and first evidence for this derived from the lack of ER tubules in dendritic spines in neurons of “dilute” mice and rats, which carry null mutations in the gene encoding for the heavy chain of myosin Va (Dekker-Ohno et al., 1996; Takagishi et al., 1996). Myosin-Va was later revealed to be involved in the translocation of ER into dendritic spines of Purkinje cells and experiments in wildtype and dilute mice support the model that myosin Va uses the actin cytoskeleton for short-range ER transport (Wagner et al., 2011). In a very recent study in CA1 pyramidal cells, expression of a dominant negative mutant for myosin Va reduced the number of spines that contain ER and enhanced mGluR-dependent LTD, which suggests that myosin Va is a key regulator of selective transport of ER tubules into highly active spines that regulates synaptic plasticity (Perez-Alvarez et al., 2020). Another recent study revealed that myosin Va-dependent ER transport into dendritic spines can be finely orchestrated by an intricate interaction between two Ca^2+^ binding proteins, calmodulin and caldendrin (Konietzny et al., 2020). However, how the actin-based myosin Va motor protein interacts with ER tubules remains to be elucidated. The ER can also dynamically move in and out of dendritic spines from hippocampal neurons and thereby regulate Ca^2+^ levels important for mGluR-dependent LTD (Toresson and Grant, 2005; Holbro et al., 2009). Treatment of Purkinje cells with low dose nocodazole suggested that the MT-dependency of ER transport into dendritic spines is minor (Wagner et al., 2011). The MT cytoskeleton is also known to transiently enter dendritic spines of hippocampal neurons (Hu et al., 2008; Jaworski et al., 2009). Although MTs are required for long-range transport of ER tubules into dendrites in hippocampal neurons (Farias et al., 2019), it remains unknown whether actin and MT crosstalk can coordinate the entrance of ER tubules into dendritic spines of these neurons.

#### The Influence of the Cytoskeleton on Mitochondria Dynamics

Mitochondria are the production house for meeting the energy demands of cells and can form a dynamic tubular network. Mitochondria are also responsible for other cellular processes including calcium homeostasis, cell differentiation, apoptosis and metabolic signaling (Nunnari and Suomalainen, 2012). Mitochondrial morphology has to be maintained for its proper functioning and this is mediated by mitochondrial fusion, fission and motility. Whilst in simple eukaryotes such as budding yeast, actin can regulate the transport of mitochondria, in animal cells, mitochondria use motor protein-coupled transport along MTs for long-distance travels while actin filaments ensure short-distance transport of mitochondria (Wu et al., 2013). Transport of mitochondria is especially essential in neurons, where the proper distribution of proteins and organelles in the dendrites and long axon requires long-distance travels. The kinesin-1 motor protein can carry mitochondria along MTs toward MT plus ends whilst dynein and its partner dynactin can transport mitochondria along MTs toward MT minus ends (Pilling et al., 2006). The coupling of mitochondria to the MT-based motor proteins kinesin-1 and dynein is mediated by the TRAK/Miro motor adaptor complex (van Spronsen et al., 2013).

Besides motility, the cytoskeleton is also involved in mitochondrial anchoring. Mitochondria can be tethered to the actin cytoskeleton, which in neurons is crucial for mitochondrial anchoring at axonal presynaptic sites (Chada and Hollenbeck, 2004; Gutnick et al., 2019). In addition, the MT-binding protein syntaphilin was discovered as a regulator of mitochondrial anchoring at presynaptic sites (Chen and Sheng, 2013). This suggests there is likely a dual role for both the actin and MT cytoskeleton in mitochondrial anchoring at presynaptic sites. However, the proteins involved in tethering mitochondria to the actin cytoskeleton and the molecular mechanisms underlying this anchoring remain to be elucidated.

Studies performed in the amoeba *Dictyostelium discoideum* showed that MTs are highly involved in mitochondrial dynamics (Woods et al., 2016). Treatment with nocodazole to depolymerize MTs led to a reduction of motile mitochondria as well as a reduced number of mitochondrial fusion and fission events. Treatment with latrunculin, which depolymerizes actin filaments, did not affect the fusion, fission or motility of mitochondria but did decrease the number of motile mitochondria. This suggests that MTs are important for mitochondrial dynamics in *Dicytostelium discoideum* whilst actin is more important for determining the percentage of moving mitochondria (Woods et al., 2016).

Mehta et al. (2019) suggested that an interaction between dynamic MTs and mitochondria in budding yeast causes an increase in mitochondrial fission events. Deletion of several kinesin-like proteins to perturb MT dynamics revealed that long and stable MTs inhibit mitochondrial fission by hampering the ring assembly of GTPase dynamin-related protein Dnm1 (Drp1 in mammals) around mitochondria because long and stable MTs introduce a physical restriction. However, depolymerized and dynamic MTs led to an increase in mitochondrial fission events which are normally essential for independent segregation of mitochondria to daughter cells during mitosis and for eliminating damaged and fragmented mitochondrial content via mitophagy (Mehta et al., 2019).

In addition, intermediate filaments have been shown to associate with mitochondria and regulate their distribution and metabolic functions (Etienne-Manneville, 2018). Intermediate filaments may be involved in positioning of mitochondria to locations with high energy demand by providing an anchoring structure. In giant axonal neuropathy patient-derived fibroblasts, mitochondrial motility is inhibited, which is linked to abnormal organization and turnover of the intermediate filament protein vimentin (Lowery et al., 2016). On the other hand, Rac1 and its effector PAK1 can phosphorylate vimentin at mitochondrial binding sites which leads to the release of mitochondria, which then gain a higher motility and lower mitochondrial membrane potential (Matveeva et al., 2015). A recent study also revealed that mutations in another intermediate filament protein, desmin, causes mitochondrial dysfunction and alterations in mitochondrial network morphology although the underlying molecular mechanisms are still unclear (Smolina et al., 2020). It would be interesting to investigate how MTs, actin and intermediate filaments coordinate their functions to ensure proper mitochondria dynamics and positioning.

### The Influence of Motile Membrane-Bound Organelles on Cytoskeletal Organization

Spatiotemporal regulation of cytoskeletal organization is essential for various cellular functions, such as the transport and dynamics of organelles and cell morphology. In the last 10 years, evidence has emerged supporting the notion of an intricate interplay between organelles and the cytoskeleton in which organelle dynamics can reciprocally regulate cytoskeletal organization. The simplest notion that motor-driven transport itself can act as an active force causing buckling and oscillation on MTs, highlights a complex mechanical interplay between cytoskeleton and motor-dependent organelle movement (Kulic et al., 2008; Man and Kanso, 2019). In addition to this mechanical interplay, organelles such as Golgi outposts, endosomes, mitochondria, and the ER, have all been implicated in cytoskeletal remodeling, specifically in MT dynamics.

Besides the perinuclear Golgi apparatus, well-known because of its role in acentrosomal MT nucleation (reviewed in Akhmanova and Steinmetz, 2019), small and more motile Golgi outposts (also referred to as satellite Golgi or Golgi vesicles) have also been found to nucleate MTs far from the nucleus in differentiated cells such as neurons, muscle cells and oligodendrocytes. In *Drosophila* and mammalian neurons, Golgi outposts distributed in dendrites can mark the main site of acentrosomal MT nucleation (Figure 1B). The Golgi structural protein GM130, as well as recruitment of γ-tubulin and CP309, the *Drosophila* homolog of AKAP450, to Golgi outpost, have been shown to be required for Golgi outpost-dependent acentrosomal MT growth and dendritic branch formation in *Drosophila* neurons (Ori-McKenney et al., 2012; Zhou et al., 2014). Golgi outposts in mouse skeletal muscle fibers were found to recruit γ-tubulin and pericentrin to sites of acentrosomal MT growth, but it remains largely unexplored whether Golgi outpost-dependent acentrosomal MT nucleation has a role in the complex MT grid-like network organization of muscle fibers (Oddoux et al., 2013). Golgi outpost have also recently been found along primary, secondary and tertiary processes of mouse oligodendrocytes, far away from the perinuclear area. The protein TPPP, recently identified in oligodendrocyte Golgi outposts, nucleates MTs in *in vitro* assays, contributes to MT branching and mixed MT polarity in 3D microfiber cultures, and is required for myelin sheath elongation *in vivo* (Fu et al., 2019).

Although two studies have implicated Golgi outposts in dendritic MT nucleation (Ori-McKenney et al., 2012; Zhou et al., 2014), other reports have challenged these results, as dragging Golgi outpost away from dendrites using an activated kinesin, did not alter γ-tubulin distribution in *Drosophila* neurons and branching points lacking Golgi outposts still contained γ-tubulin (Nguyen et al., 2014). More recent work showed that these γ-tubulin nucleation sites in dendritic branches colocalize with and are regulated by components of the canonical *Wnt* signaling pathway. Puncta containing *Wnt* signaling components were found to colocalize with Rab5-positive early endosomes, from which new MT growth initiated (Figure 1B). This evidence suggests the involvement of early endosomes in dendritic acentrosomal MT nucleation (Weiner et al., 2020). However, more experiments are required to evaluate whether early endosome positioning in dendritic branching points are required for MT nucleation and branch formation. It would be interesting to evaluate whether the removal of early endosomes and other organelles from dendrites alters γ-tubulin distribution and local MT dynamics.

Mitochondria have also been implicated in MT growth (Figure 1B). In *Drosophila* spermatids, a non-centrosomal, testes-specific isoform of centrosomin is localized to giant mitochondria, and it is required to recruit the MT nucleator γ-tubulin ring complex (γ-TuRC) to regulate MT assembly and spermiogenesis (Chen et al., 2017).

Finally, an important role for ER – MT interactions in MT organization has been suggested for years. Mutations in the ER-associated proteins Spastin, Atlastin-1 and REEP1 cause the neurodegenerative disease HSP, which is characterized by progressive spasticity in lower motor neurons (Blackstone, 2012). The MT-severing protein Spastin, GTPase Atlastin-1 and MT-interacting protein REEP1 form a complex that interacts with MTs and has been shown to be enriched in the axonal ER in rat cortical neurons (Park et al., 2010). Although the role of these proteins in maintaining the ER tubule network has been shown (reviewed by Goyal and Blackstone, 2013), the exact role of axonal ER tubules in MT organization has been less explored. More recent experiments in rat hippocampal neurons have demonstrated an important role for ER tubules in MT stabilization and axon formation. ER tubules are preferentially distributed in the growing axon of developing neurons and interact with and stabilize MTs. Controlled removal of ER tubules from the axon, by coupling the ER to a minus-end directed motor, causes MT instability and prevents elongation of the developing axon (Farias et al., 2019). In addition, the Atlastin-1 ortholog, Atln-1, which is distributed in dendrites of *C. elegans* PVD sensory neurons, has been found to colocalize with and be required for MT entrance into secondary and tertiary dendritic branches (Liu et al., 2019). Although some evidence has started to emerge regarding the role of the ER in MT dynamics, it still remains unclear whether different domains of the ER play different roles in MT organization.

### Complex Inter-Organelle Interactions in Coordination With the Cytoskeleton

It is now well-established that organelles interact with each other at MCSs and knowledge on the types and importance of MCSs has greatly increased in the past few years. Since the ER is the largest organelle and forms a continuous and interconnected network throughout a cell, it is not surprising that it participates in most MCSs (reviewed in Phillips and Voeltz, 2016). The ER can interact with several other organelles including mitochondria, late endosomes/lysosomes, Golgi, and the plasma membrane. These interactions are involved in organelle positioning and fission, and lipid and Ca^2+^ homeostasis (Phillips and Voeltz, 2016; Allison et al., 2017; Fowler et al., 2019). However, the interplay between inter-organelle contacts and the cytoskeleton is less well-studied. In this section, we highlight the current knowledge on the complex interactions between membrane-bound organelles in coordination with the cytoskeleton and emphasize the importance of this relationship in organelle dynamics including fission, fusion and transport.

#### Membrane-Bound Organelles Can “Hitchhike” for Transport

The concept of hitchhiking of membrane-bound organelles on other organelles for their transport emerged just recently. In the fungus *Ustilago Maydis*, movement of the ER, lipid droplets and peroxisomes is mediated by motile Rab5-positive early endosomes via kinesin-3 and dynein on MTs (Guimaraes et al., 2015; Salogiannis and Reck-Peterson, 2017). In mammals, several examples of this type of organelle hitchhiking have also been recently discovered. For instance, it was found that the ER can tether itself onto late endosomes/lysosomes for hitchhiking in cell lines and neurons (Guo et al., 2018; Lu et al., 2020). GI-SIM imaging also revealed that mitochondria can hitchhike on motile late endosomes/lysosomes for their translocation along MTs (Guo et al., 2018). This hitchhiking mechanism will be discussed in more detail below.

#### Complex Interactions Between the ER, Late Endosomes/Lysosomes and the MT Cytoskeleton

The ER can form extensive and dynamic contacts with late endosomes/lysosomes at MCSs. In fact, almost all late endosomes/lysosomes were found to be in contact with the ER (Friedman et al., 2013; Guo et al., 2018). MCSs between the ER and late endosome/lysosomes are involved in many essential cellular tasks including endosome fission and positioning. 3D reconstructions from electron microscopy serial tomography in COS7 cells and live-cell imaging revealed that MCSs between the ER and endosomes are tightly associated with MTs and this association can be maintained when endosomes are moving along MTs (Friedman et al., 2013). GI-SIM imaging revealed that the ER can rearrange its coral large tubular network into smaller structures that tightly wrap around late endosomes/lysosomes where they are close to MTs (Figure 1C). Late endosomes/lysosomes that are not in contact with the ER were observed to undergo diffusive motion along MTs suggesting that the ER coordinates the stabilization of late endosomes/lysosomes (Guo et al., 2018). Interestingly, it has been previously shown that the ER can mediate kinesin-1 coupling to late endosomes/lysosomes for transport to the cell periphery via motor protein transfer (Raiborg et al., 2015). In this intriguing mechanism, the ER-resident protein Protrudin was shown to transfer kinesin-1 to the adaptor protein Rab7 and its effector FYCO1 localized on late endosomes/lysosomes for their anterograde transport along MTs in human and rat cell lines (Raiborg et al., 2015). This ER-mediated late endosome/lysosome translocation to the cell periphery was shown to promote neurite outgrowth in the neuroendocrine cell line PC12 (Raiborg et al., 2015). In a recent study, an interplay between the ER, lysosomes and MTs was proposed to play a role in lysosome translocation into the axon of rat hippocampal neurons (Özkan et al., 2020). Disruption of somatic, but not axonal ER tubules led to an accumulation of enlarged and less motile lysosomes in the soma, thus triggering a drastic reduction in the translocation of lysosomes into the axon. A similar phenotype was also observed after the knockdown of another ER-resident protein P180, which has binding domains for both MTs and kinesin-1. P180 was found to be particularly enriched in a region preceding the axon initial segment called the pre-axonal region. Serial z-stacking of this region revealed that P180-enriched ER can undergo ring rearrangements to tightly wrap around lysosomes. Lysosomes contacting the ER were observed in close proximity to stable MTs decorated by a rigor kinesin-1 motor mutant. Together, this raises the possibility that P180 stabilizes the interaction between MTs and ER-late endosome/lysosome contacts via its MT-binding domain and loads kinesin-1 onto late endosomes/lysosomes thereby facilitating their translocation into the axon (Özkan et al., 2020).

The first evidence revealing a role for ER-endosome contacts in endosomal fission came from studies in COS7 cells in which contacts between the ER and early endosomes or late endosomes/lysosomes defined the endosomal constriction and fission site (Rowland et al., 2014). More recent evidence showed that the ER-resident protein Spastin and the endosomal protein IST1 interact with each other at ER-endosome contact sites to promote endosomal tubule fission in mammalian cell lines (Allison et al., 2013, 2017). Primary cortical neurons from a Spastin-HSP mouse model and IPSC-derived HSP patient neurons contained abnormal enlarged lysosomes, which suggests that disruption of Spastin in ER-endosome contact sites impacts lysosome size in neurons (Allison et al., 2017). However, how the ER regulates lysosome size remained elusive. Interestingly, direct contacts between the ER and late endosomes/lysosomes have been observed in hippocampal neurons, and this interaction is required for late endosome/lysosome fission. Late endosomes/lysosomes undergo several fusion and fission events in control neurons, while ER tubule disruption causes enlarged lysosomes with reduced fission capacity (Özkan et al., 2020).

The role of the ER in late endosome/lysosome transport or fission has often been studied separately. Just recently, Özkan et al. (2020), proposed a model in which these events are connected through a complex interaction between MTs and ER-lysosome contacts that promotes lysosome fission and subsequent lysosome transport into the axon (Figure 1D).

Whilst the ER is now well-established as a prominent regulator of lysosome transport and fission, the effect of lysosome positioning on ER tubule rearrangement has remained unclear. With GI-SIM, the ER was observed to hitchhike on moving late endosomes/lysosomes along MTs for their transport (Figure 1C; Guo et al., 2018). In addition, ER fission was observed when moving late endosomes/lysosomes collided with and broke ER tubule network at junctions between fused tubules. Guo et al. suggested that Atlastin-1, an ER-shaping protein implicated in the formation of three-way junctions could be involved in this ER fission mechanism. However, the breaking of ER tubules was also observed along a single tubule (Figure 1C; Guo et al., 2018). It remains unknown which molecules are involved in this ER-breaking process, and further investigation is required. Dual-color single particle tracking in COS7 cells revealed that 98% of lysosomes are moving simultaneously with the ER and the growing tips of the ER associated with lysosomes are elongated and merged with the existing ER network to form three-way junctions whilst the ER growth tips that are not associated with lysosomes cannot maintain elongation and are retracted (Lu et al., 2020). In addition, manipulation of lysosome positioning by coupling lysosomes to dynein for retrograde movement to the perinuclear area or kinesins for anterograde movement to the cell periphery has been recently shown to cause a reduction in ER tubules or extension of ER tubules to the periphery of cells, respectively (Lu et al., 2020). Moreover, live-cell imaging in cultured *Xenopus laevis* retinal ganglion cell (RGC) axons showed that the ER can rearrange its structure into a ring arrangement that tightly wraps around lysosomes. These lysosomes then pull out the ER tubule for its extension. This suggests that ER tubule elongation is driven by lysosome positioning and is essential for ER tubule remodeling in axons to support axonal growth (Lu et al., 2020).

All this evidence indicates the importance of the interplay between the ER and lysosomes where the ER modulates lysosome fission and translocation, and lysosome motility mediates ER tubule transport and remodeling.

Defects in ER morphology and function as well as an impairment in the endolysosomal system are key pathological features of neurological diseases such as HSP. Therefore, it will be crucial to identify the regulators and molecular mechanisms underlying the crosstalk between these two organelles and further establish the link between these organelles and the cytoskeleton.

#### Complex Interactions Between Mitochondria, the ER and the Cytoskeleton

Friedman et al. (2011) revealed that ER-mitochondria contact sites are mainly formed at a pre-constriction site for mitochondrial fission, suggesting that contacts between the ER and mitochondria regulates mitochondrial fission (Figure 1D). The recruitment of Drp1 to the pre-constriction site is the key regulatory step in mitochondrial fission (Smirnova et al., 2001). However, other studies revealed that pre-constriction site formation can be mediated by Drp1-independent mechanisms (Korobova et al., 2013). Hatch et al. suggested a possible mechanism in which the ER-driven interaction between mitochondria and the cytoskeleton regulates mitochondrial fission in mammalian cells by formation of a pre-constriction site and subsequent recruitment of Drp1 (Korobova et al., 2013; Hatch et al., 2014). ER-mitochondria contact sites marking the putative pre-constriction site can recruit formin-like protein INF2 which triggers actin polymerization on the ER. This actin polymerization then generates forces on the mitochondrial membrane that leads to the formation of a pre-constriction site to which Drp1 is recruited. The accumulation of Drp1 and subsequent GTP hydrolysis drives constriction and scission of the membrane leading to mitochondrial fission (Korobova et al., 2013; Hatch et al., 2014). Lee et al. (2016), proposed a model that includes a role for dynamin 2 (Dyn2) in the final step of mitochondrial fission. Drp1-mediated constriction of the mitochondrial membrane promotes Dyn2 assembly at the constriction site where it can promote membrane fission for successful mitochondrial division. Live-cell imaging and electron microcopy in different mammalian cells lines suggested Dyn2 and Drp1 work in harmony in sequential constriction steps to promote mitochondrial fission (Lee et al., 2016). By contrast, a recent study using human fibroblast cell lines showed that the knockout of Dyn1, 2, and 3 or knockdown of only Dyn2 did not alter mitochondrial fission. Similar results obtained in HeLa cells lacking Dyn1, 2, and 3 showed no effect on mitochondrial fission, eliminating the possibility of a cell-type specific effect of Dyn2 on mitochondrial fission (Fonseca et al., 2019). These controversial results request further studies to determine whether or not Dyn2 is dispensable for the scission of the mitochondrial membrane.

Recent studies revealed a more complex mechanism rather than simple Drp1-mediated mitochondrial fission. This mechanism involves an interplay between mitochondria, the ER, INF2-driven actin polymerization and calcium uptake by mitochondria (Chakrabarti et al., 2018; Steffen and Koehler, 2018). Extracellular calcium influx induces INF2-mediated actin polymerization on the ER that triggers subsequent calcium uptake by mitochondria from the ER. Calcium uptake by the inner membrane of mitochondria through the mitochondrial calcium uniporter drives inner membrane scission that precedes outer membrane fission, suggesting INF2-mediated actin polymerization on the ER regulates mitochondrial fission by affecting mitochondrial calcium uptake (Chakrabarti et al., 2018; Steffen and Koehler, 2018).

Another intriguing study investigating the role of ER morphology on mitochondria dynamics in *C. elegans* PVD neurons revealed an interaction between the ER network and mitochondria at dendritic branching points, which is crucial for mitochondrial fission (Liu et al., 2019). They showed that mitochondria are highly enriched at the dendritic branch points where the ER network is more complex in neurons expressing wildtype Atlastin-1. However, neurons expressing mutant Atlastin-1 had a less complex ER network and a reduced number of mitochondria at dendritic branch points (Liu et al., 2019). This suggests that the local ER network regulates mitochondrial distribution at dendritic branch points. Analysis of mitochondrial fission showed that the local ER network at branch points can attach to and promote the fission of mitochondria. Together with unpublished results indicating that ER tubule extension strongly relies on transient MT entry into dendrites, Liu et al. proposed a model in which the interaction between the complex ER network and mitochondria at dendritic branch points depends on MTs and is required for mitochondrial fission (Liu et al., 2019).

Surprisingly, a recent study revealed that ER-mitochondria contacts can also mark the mitochondrial fusion site (Abrisch et al., 2020) consistent with the observation that mitochondria fusion occurs in proximity to the ER (Guo et al., 2018). Abrisch et al. found that Mitofusin-1 (Mfn1), a regulator of mitochondrial fusion, is localized to ER-mitochondria contact sites and fluorescence loss in photobleaching (FLIP) experiments in U2OS and COS7 cells showed that Mfn1 and Drp1 colocalize and both fusion and fission events take place at the same site where the ER contacts mitochondria (Abrisch et al., 2020). This suggests a remarkable mechanism in which the ER can regulate both mitochondrial fusion and fission at the same position and provides a way to respond to cellular needs in a quick and coordinated manner. However, how switching between fusion and fission is achieved remains unclear.

#### Complex Interactions Between Mitochondria, Late Endosomes/Lysosomes, and the MT Cytoskeleton

Mitochondria can also hitchhike on motile late endosomes/lysosomes coupled to MT-bound motor proteins. Late endosomes/lysosomes that move in close proximity to mitochondria were reported to be important for mitochondrial morphology. Dynamic tubulation of mitochondria can be achieved during this hitchhiking, thus providing a mechanism for mitochondrial fusion (Guo et al., 2018). An indirect involvement of the MT cytoskeleton in the changes of mitochondrial morphology and corresponding functions can be speculated as mitochondrial hitchhiking along motile late endosomes/lysosomes requires MTs.

In summary, there is an outstanding communication between membrane-bound organelles in association with cytoskeleton, which is essential for organelle positioning, dynamics and morphology as well as cytoskeletal dynamics.

## The Interplay Between the Cytoskeleton and Biomolecular Condensates

Biomolecular condensates form distinct compartments in the cell that lack a membrane boundary and are present in both the cytoplasm and nucleoplasm (Banani et al., 2017; Boeynaems et al., 2018; Sabari et al., 2020). In the cytoplasm, various biomolecular condensates have been identified of which some are cell-type specific (e.g., germ granules in germline cells) or stress condition-dependent (e.g., Sec bodies and stress granules) (Voronina et al., 2011; Decker and Parker, 2012; Zacharogianni et al., 2014; Banani et al., 2017). Liquid-liquid phase separation can also underlie the formation of neuronal synaptic densities and membrane clusters near the plasma membrane (Wu X. et al., 2020). In addition to this, a fast-growing body of work shows that a number of viruses form cytosolic liquid-liquid phase separated condensates upon infection of cells, termed “viral factories” and viruses are known to induce changes in stress granule and P-body formation and composition (Alberti and Dormann, 2019).

Several cytosolic biomolecular condensates interact with the cytoskeleton, either directly or indirectly via “hitchhiking” (see section “Interactions Between Membrane-Bound Organelles and Biomolecular Condensates in Conjunction With the Cytoskeleton”) and/or influence cytoskeletal remodeling. In this section we will focus on several of the best-studied cytoplasmic biomolecular condensates (P-bodies, stress granules and RNP transport granules) and discuss their direct interactions and interplay with the cytoskeleton as well as highlight the known functional roles of these interactions.

### Direct Interactions Between Biomolecular Condensates and the Cytoskeleton

#### Regulation of Biomolecular Condensate Transport by the Cytoskeleton

**FIGURE 2:**
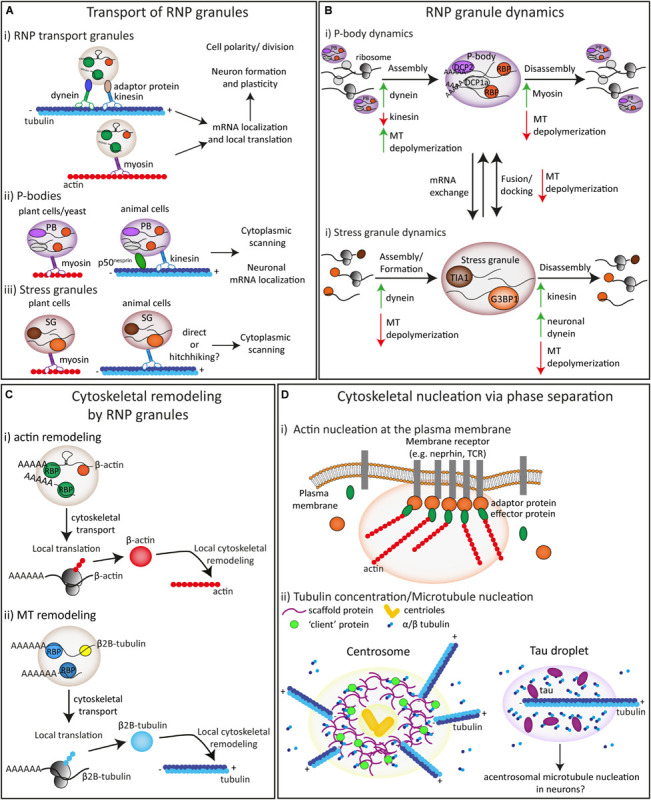
The interplay and interactions between biomolecular condensates and the cytoskeleton. **(A)** The transport of RNP transport granules (i), P-bodies (ii) and stress granules (iii) is mediated by the actin and microtubule cytoskeleton driven by motor proteins. **(B)** The formation/assembly, disassembly and the fusion/docking of P-bodies (i) and stress granules (ii) is influenced by the cytoskeleton and motor proteins. **(C)** The local cytoskeleton can be remodeled by RNPs through mRNA supply and local translation of both actin (i) and microtubule (ii) proteins. **(D)** Both actin (i) and microtubule (ii) nucleation can be driven by concentration of specific proteins via phase separation at specific sub-cellular locations.

Like membrane-bound organelles, biomolecular condensates can use the cytoskeleton for active, motor-driven transport throughout the cell (Figure 2A). One of these biomolecular condensates is the RNP transport granule, which consist of RNA-binding proteins (RBPs) and messenger RNAs (mRNAs). These granules can form by liquid-liquid phase separation and are well-known to be transported to ensure proper mRNA localization to specific subcellular locations for subsequent local mRNA translation (Figure 2A; Knowles et al., 1996; Buxbaum et al., 2015; Das et al., 2019). This RNP transport for mRNA localization is highly conserved and has been observed in many different model systems such as budding yeast (*Saccharomyces cerevisiae*), fibroblasts, *Drosophila* and *Xenopus* oocytes, oligodendrocytes and neurons where it plays a crucial role in biological processes such as cell division, migration, cell polarization, and axon guidance and neuronal synaptic plasticity (Holt and Bullock, 2009; Buxbaum et al., 2015). Extensive research in these different organisms and cell types has shown that mRNA/RNP transport depends on both the microtubule and actin cytoskeleton and is driven by kinesin, dynein and myosin motor proteins (Figure 2A; Kanai et al., 2004; Gopal et al., 2017; Mofatteh and Bullock, 2017; McClintock et al., 2018; Das et al., 2019; Baumann et al., 2020). RNA-binding proteins can interact with molecular motor proteins thereby enabling the transport of translationally repressed mRNAs in RNP granules along the cytoskeleton. During cell division of budding yeast, a well-characterized mRNP granule containing *Ash1* mRNA and several other mRNAs is transported to the tip of a daughter cell on the actin cytoskeleton mediated by the adaptor protein She3p, that couples the RNA-binding protein (RBP) She2P with the myosin motor protein Myo4p (Long et al., 1997; Takizawa et al., 1997; Edelmann et al., 2017). Other well-established examples include the transport of *Vg1* mRNA in *Xenopus* oocytes, that relies on overlapping functions of both kinesin-1 and kinesin-2 motors (Messitt et al., 2008) and the MT-based transport and actin-mediated anchoring of *oskar* and *bicoid* mRNAs in Drosophila oocytes (Trcek and Lehmann, 2019). In addition, several recent studies, both *in vitro* and in rat DRG neurons and mouse hippocampal neurons, have elucidated the interaction of the several RBPs (APC, SFPQ, and ZBP1) with specific adaptor and motor proteins, such as KAP3 with kinesin-2 and KLC1 or PAT1 with kinesin-1 (Baumann et al., 2020; Fukuda et al., 2020; Wu H. et al., 2020). Despite this progress in our understanding of RNP granule transport, many questions remain unanswered; for example, it is unclear how anchoring of RNP granules to specific subcellular locations is regulated and how mRNAs and RBPs are released from motor proteins.

In addition to RNP transport granules, the cytoplasm contains several other well-studied membraneless RNP granules such as P-bodies and stress granules (Buchan, 2014; Riggs et al., 2020). P-bodies are RNP granules that are highly conserved from yeast to humans (Eystathioy et al., 2002; Sheth and Parker, 2003; Riggs et al., 2020). In mammalian cells, P-bodies are present under basal conditions but can dynamically change in number, size and its components upon changes in the availability of non-translating mRNAs (Kedersha et al., 2005; Teixeira et al., 2005; Decker and Parker, 2012; Wang et al., 2018). Stress conditions that affect the amount of non-translating mRNAs therefore change P-body number and size. P-bodies are enriched in translationally inactive mRNAs and proteins involved in mRNA decapping, turnover and silencing, but lack ribosomal proteins and translation initiation factors (Hubstenberger et al., 2017; Luo et al., 2018). They have therefore been suggested to play a role in translational repression, microRNA-induced mRNA silencing, RNA storage and mRNA decay, although recent studies suggest that decay may not occur in P-bodies and they mainly serve as a repository for translationally repressed mRNAs that can reenter translation upon release (Brengues et al., 2005; Horvathova et al., 2017; Hubstenberger et al., 2017; Riggs et al., 2020).

P-bodies are able to perform directed movement in plant, yeast and mammalian cells (Figure 2A; Kedersha et al., 2005; Aizer et al., 2008; Cougot et al., 2008; Hamada et al., 2012; Garmendia-Torres et al., 2014; Rajgor et al., 2014). In plant cells (*Arabidopsis thaliana*), long-distance movement of P-bodies was dependent on the actin cytoskeleton and pausing behavior was observed at cortical microtubules (Figure 2A; Hamada et al., 2012; Steffens et al., 2014). In mammalian cells, live-cell imaging and single particle tracking experiments showed that P-bodies associate to both the actin and MT cytoskeleton (Aizer et al., 2008). Stationary P-bodies were associated with actin whereas P-bodies exhibited mainly confined movements and occasional long-range movements in association with MTs (Figure 2A; Aizer et al., 2008; Rajgor et al., 2014). MT depolymerization by nocodazole or vinblastine treatment severely reduced P-body movement (Aizer et al., 2008).

Several proteins have been implicated in the interaction between P-bodies and the cytoskeleton. Early studies in yeast showed that P-body markers Edc3p and Dcp1p can interact with tubulin proteins (Gavin et al., 2006). In addition, several P-body components, as well as mRNAs, were shown to co-precipitate and colocalize with the myosin motor protein Myo2p in budding yeast (Chang et al., 2008). During cell division in yeast, unidirectional transport of P-bodies, marked by Edc3p, was observed to be dependent on a complex of Myo4p and the She2P/She3P RBPs (Figure 2A; Garmendia-Torres et al., 2014). In HeLa cells, the expression of the dominant negative tail of another myosin protein, Myosin Va, reduced P-body motility although the authors suggested that this could be caused indirectly by reducing mRNP transport toward P-bodies (Lindsay and McCaffrey, 2011). Another study showed that interactions between DCP proteins and myosin proteins are likely conserved in yeast (Dcp1p with myo2p) and mammals (human Dcp1a and Dcp1b with mouse myosin Va) by performing yeast two-hybrid and coprecipitation experiments (Steffens et al., 2014). The presence of several myosin proteins in a large screen identification of P-body components further confirm the association between myosin motor proteins and P-bodies in mammalian cells (Hubstenberger et al., 2017). A more direct and functional link was found with the identification of an isoform of the MT-associated protein Nesprin, p50^*nesp*1^. This protein was found to interact with and localize to P-bodies and anchor them to MTs, thereby facilitating P-body movement (Figure 2A; Rajgor et al., 2014). In neurons, several reports show that P-bodies or P-body-like structures that partly resemble neuronal transport granules undergo motor-driven transport on MTs in dendrites (Cougot et al., 2008; Zeitelhofer et al., 2008; Oh et al., 2013). These neuronal P-bodies increase their localization to dendritic synapses in response to neuronal activation, driven by the kinesin-1 motor (Figure 2A; Zeitelhofer et al., 2008; Cougot et al., 2008; Oh et al., 2013). Neuronal P-bodies have also been observed in axons of peripheral neurons but whether and how they are actively transported remains to be investigated (Melemedjian et al., 2014; Sahoo et al., 2018).

Stress granules are closely related to both P-bodies and RNP transport granules and can share many of the same components but appear only after stress conditions that inhibit translation (Kedersha et al., 2005; Khong et al., 2017; Markmiller et al., 2018; Youn et al., 2018; Matheny et al., 2019; Youn et al., 2019). Their exact composition differs between specific stress conditions and cell types (Markmiller et al., 2018). Stress granules sequester mRNAs and stalled initiation complexes and are therefore considered to be a storage site for certain non-translating mRNAs during stress that can re-enter translation when stress is relieved (Riggs et al., 2020). A recent study, however, partly challenged this notion and showed that stress granules also contain translating mRNAs that can shuttle between stress granules and the cytoplasm (Mateju et al., 2020).

Several studies have shown that stress granules can undergo directed transport although they appear to be less motile than P-bodies (Figure 2A; Kedersha et al., 2005). In plant cells, one study observed rapid and long-distance movement of stress granules marked by eIF42-GFP that decreased only upon actin, but not MT depolymerization (Figure 2A; Hamada et al., 2018). By contrast, another study in plant cells observed movement of stress granules (marked by Rbp47b) along MTs but the role of actin was not examined (Gutierrez-Beltran et al., 2015). In HeLa cells, it was shown that arsenite-induced stress granules move throughout the cytoplasm in a mostly diffusive manner (Chernov et al., 2009; Nadezhdina et al., 2010). However, a small percentage (±10%) of stress granules exhibited directed movement (Figure 2A; Nadezhdina et al., 2010). Both diffusive and directed stress granule movement was dependent on MTs, but not actin, as shown by colocalization experiments and treatment with cytoskeleton destabilizing drugs. It remains unknown which proteins drive stress granule movement, and it is unclear if movement can occur via direct interactions or whether it is mainly driven by indirect interactions with MTs via “hitchhiking” mechanisms (Liao et al., 2019), which will be further discussed in section “Interactions Between Membrane-Bound Organelles and Biomolecular Condensates in Conjunction With the Cytoskeleton.”.

#### Regulation of Biomolecular Condensate Remodeling by the Cytoskeleton

Besides their movement throughout the cell, RNP granules (transport/neuronal granules, P-bodies and stress granules) are known to be highly dynamic structures that can assemble, disassemble and remodel by rapidly exchanging mRNAs and possibly proteins with each other and the cytoplasm (Kedersha et al., 2005; Aulas et al., 2017; Wang et al., 2018; Moon et al., 2019). Fluorescence recovery after photobleaching (FRAP) experiments have shown that RNP granules can exchange protein components with the cytoplasm (Tauber et al., 2020). Various molecular mechanisms such as extracellular cues, signaling, protein-protein interactions and posttranslational modifications as well as cellular states are known to influence these dynamics (Formicola et al., 2019; Tauber et al., 2020). In addition to this, the cytoskeleton plays an important role in the remodeling of biomolecular condensates (Figure 2B).

P-body formation and disassembly/dynamics are also affected by the cytoskeleton and motor-based transport. In budding yeast, U2OS and HeLa cells, depolymerization of MTs with different treatments led to an increase in P-body formation (Figure 2B; Sweet et al., 2007; Aizer et al., 2008; Carbonaro et al., 2011; Huang et al., 2011; Ayache et al., 2015). Interestingly, taxol-induced MT stabilization also increased P-body number (Carbonaro et al., 2011). The mechanisms that lead to increased P-body formation after MT depolymerization or stabilization are not entirely clear, but these MT disruptions likely lead to a decrease in mRNA translation and a release of mRNAs from polysomes. However, in yeast, benomyl-induced MT depolymerization did not seem to affect mRNA translation based on polysome profiles (Sweet et al., 2007). On the other hand, it has been shown that MT depolymerization with both nocodazole and vinblastine treatment decreases protein synthesis (Carbonaro et al., 2011; Coldwell et al., 2013; Ayache et al., 2015; Szaflarski et al., 2016). It is possible that both MT-depolymerization and stabilization (through impaired MT dynamics) lead to defective mRNA transport (Lifland et al., 2011; Pease-Raissi et al., 2017), either to sites of active translation or possibly into P-bodies themselves. In support of this, both MT stabilization with taxol or MT depolymerization with vinblastine or 2ME2 induced the release of HIF-1α mRNA from polysomes and subsequent incorporation into P-bodies. This effect was reversible after nocodazole washout and subsequent MT repolymerization (Carbonaro et al., 2011). In contrast to mRNAs, the depolymerization of MTs with nocodazole did not change the dynamic exchange of Dcp proteins in and out of P-bodies based on FRAP experiments (Aizer et al., 2008).

The involvement of specific motor proteins on P-body formation has also been investigated (Loschi et al., 2009). Whilst under basal conditions P-body size or number was not affected by knockdown of the motor proteins dynein and kinesin, their increase in size was significantly affected after dynein, but not kinesin-1 knockdown under ER or oxidative stress conditions (Loschi et al., 2009). Interestingly, simultaneous knockdown of kinesin abrogated the effect of dynein knockdown on the stress-induced increase in P-body size. This led the authors to suggest that both anterograde and retrograde transport are important for the exchange of mRNAs from RNP granules into and out of P-bodies, at least under stress conditions (Figure 2B; Loschi et al., 2009). In addition to this, siRNA-mediated knockdown of the actin-based Myosin Va motor was found to decrease P-body assembly, but not stress granule formation in HeLa cells (Figure 2B; Lindsay and McCaffrey, 2011). The release of mRNAs from P-bodies may indeed also be regulated by the actin cytoskeleton, since P-body disassembly was found to be delayed in a yeast myosin motor mutant (Chang et al., 2008).

Under stress conditions, P-bodies often transiently dock in close proximity to stress granules and mRNAs are able to move bidirectionally between P-bodies and stress granules (Kedersha et al., 2005; Moon et al., 2019). When P-bodies and stress granules are docked together, they appear less motile (Kedersha et al., 2005) and the close docking of stress granules with P-bodies is dependent on intact microtubules (Figure 2B; Aizer et al., 2008; Rajgor et al., 2014). In cells with intact microtubules but where P-bodies were detached from microtubules by overexpression of a p50^*nesp*1^ mutant the association between stress granules and P-bodies was also reduced (Rajgor et al., 2014). Together, this suggests that the cytoskeleton may provide a scaffold for P-body-stress granule docking. It would be interesting to determine if P-bodies and stress granules dock at specific locations on microtubules and how this could be mediated.

Stress granule formation is normally a reversible process that starts only under certain stress conditions (Wheeler et al., 2016; Riggs et al., 2020). After stress induction, granules start increasing in size by accumulating components or fusing with other stress granules (Kedersha et al., 2005; Nadezhdina et al., 2010; Wheeler et al., 2016). When stress is relieved, stress granules can disassemble or dissolve into the cytoplasm by releasing their contents into the cytoplasm. Splitting or fission of larger stress granules into smaller ones has been observed during disassembly (Kedersha et al., 2005; Nadezhdina et al., 2010; Wheeler et al., 2016; Lee et al., 2020). Both the formation and disassembly of stress granules have been shown to be dependent on the cytoskeleton and motor proteins (Figure 2B). Two initial studies first reported that MT depolymerization completely inhibited the formation of stress granules, but subsequent studies have shown it is more likely that MT disruption does not completely prevent stress granule formation but rather results in more numerous but smaller stress granules (Ivanov et al., 2003; Kwon et al., 2007; Chernov et al., 2009; Fujimura et al., 2009; Kolobova et al., 2009; Loschi et al., 2009). Interestingly, the addition of the MT-stabilizing drug taxol also resulted in smaller stress granules (Chernov et al., 2009). After stress relief, MT disruption impaired stress granule disassembly (Nadezhdina et al., 2010). The role of motor proteins in stress granule assembly and disassembly remains somewhat unclear as several studies have reported contradictory results. Whilst two studies reported a decreased formation of stress granules after treatment with dynein inhibitors (Kwon et al., 2007; Tsai et al., 2009), two other studies observed no effect (Chernov et al., 2009; Fujimura et al., 2009). In support of a role for dynein motors and retrograde transport in stress granules assembly, knockdown of dynein heavy chain 1 (DHC1) and the adaptor protein Bicaudal D1 in mammalian cells inhibits stress granule formation, and knockdown of dynein light chain subunit 2A in primary neurons impaired stress granule formation (Loschi et al., 2009; Tsai et al., 2009). Kinesin knockdown or kinesin inhibitors did not impair stress granule formation, but KIF5B or KLC knockdown did delay stress granule disassembly (Figure 2B; Loschi et al., 2009; Tsai et al., 2009). By contrast, inhibition of dynein, but not of kinesin motors in neurons, delayed stress granule disassembly (Figure 2B; Tsai et al., 2009). Interestingly, kinesin and dynein transport seemed to counterbalance each other as knockdown of KIF5B counteracted the effect of DHC1 knockdown in stress granule assembly and conversely, DHC1 knockdown partially rescued the delay in stress granule disassembly after KIF5B knockdown (Loschi et al., 2009).

Together, these results suggest microtubules are not crucial for the initial assembly of stress granules but are important for the formation of larger stress granules and for stress granule disassembly. This effect is likely mediated by facilitating transport of protein and mRNA components into and out of stress granules via motor proteins and/or increasing the chance of stress granule fusion.

Research on the role of the actin cytoskeleton on stress granule formation has yielded inconclusive results. One report observed an increase in stress granule size after Latrinculin B-mediated actin depolymerization in CV-1 cells (Ivanov et al., 2003), whilst another study found more numerous but smaller stress granules after cytochalasin B treatment in COS-7 cells (Loschi et al., 2009). Yet another study reported no change in stress granule formation after latrunculin B or cytochaslasin D treatment in HeLa cells, although stress granule size was not measured (Kwon et al., 2007). These discrepancies could be explained by differences in treatments and non-actin related side-effects (Ornelles et al., 1986). In addition, the actin-based Myosin Va motor was not required for stress granule formation, but this does not exclude the possibility that other actin-based motors are involved (Lindsay and McCaffrey, 2011).

Although understudied, several studies report an association of intermediate filaments with RNP condensates. A recent report showed that the intermediate filament protein vimentin associates with both P-bodies and stress granules and both stress granule formation and clearance is affected in vimentin knockout cells (Pattabiraman et al., 2020). Toxic ALS-associated poly-dipeptides were also found to bind along vimentin filaments (Lin et al., 2016). In addition to this, giantin-based intermediate filaments have been suggested to serve as a scaffold for cytoplasmic condensates marked by myxovirus resistance proteins (reviewed in Sehgal et al., 2020). Together this shows that intermediate filaments may play an important role in the dynamics of various biomolecular condensates.

### Biomolecular Condensates Can Influence the Cytoskeleton

Conversely, biomolecular condensates can influence the actin and microtubule cytoskeleton, either by supplying mRNAs encoding cytoskeletal proteins for local translation (Figure 2C) or by concentrating certain proteins that promote actin or microtubule nucleation (Figure 2D).

Early work has shown that mRNAs encoding cytoskeletal proteins such as Tau and β-actin are localized to specific subcellular locations in neurons, oocytes and other cell types (Jeffery et al., 1983; Litman et al., 1994). As mentioned above, these mRNAs travel together with RBPs in RNP granules along the cytoskeleton, in a translationally repressed state and are released for local translation upon stimulation with specific cues or signals. This local translation of mRNAs encoding cytoskeletal proteins is exemplified in axon guidance, where the steering of axonal growth cones in response to guidance cues relies on the local protein synthesis of specific cytoskeletal proteins (Buxbaum et al., 2015; Cioni et al., 2018). In addition, it was suggested that mRNAs encoding the actin cytoskeleton regulators Arp2 and Nd1 can be released from P-bodies in dendrites in response to neuronal activation, thereby influencing local F-actin dynamics at synapses (Oh et al., 2013). Intriguingly, it has been shown that both actin and microtubule regulators can act as RBPs in RNP granules containing mRNAs coding for cytoskeletal regulators (Preitner et al., 2014; Vidaki et al., 2017). This provides a possible self-regulatory mechanism in which certain cytoskeletal regulators coordinate the assembly of the actin or microtubule cytoskeleton by forming RNP granules that contain mRNAs encoding their components (Figure 2C).

As mentioned above, besides RNP granule formation, liquid-liquid phase separation can also underlie the formation of biomolecular condensates that enables the concentration of certain proteins to drive specific functions. In this manner, both actin and microtubule nucleation can be promoted by concentrating cytoskeletal proteins via phase separation mechanisms. For actin, this has been shown to occur near the plasma membrane, where clustering of transmembrane proteins with adaptor proteins can induce phase transitions forming a dense phase at the plasma membrane. This mechanism increases the dwell time of effector proteins that are recruited to these adaptor proteins and increase the likelihood of activation of signaling pathways (Case et al., 2019). In response to extracellular signals, the transmembrane protein Nephrin is phosphorylated and has been shown to subsequently trigger the formation of these phase-separated membrane clusters in combination with the adaptor protein Nck and the actin nucleation factors N-WASP and the ARP2/3 complex (Banjade and Rosen, 2014; Case et al., 2019; Kim et al., 2019). This promotes an increase in actin nucleation/assembly, thereby influencing the local organization of the cytoskeleton close to plasma membrane (Figure 2D). Similarly, T-cell receptors can form phase-separated clusters at the plasma membrane via phosphorylation-dependent association with the linker protein LAT (Su et al., 2016). These TCR-LAT clusters recruit specific adaptor and effector proteins whilst excluding others. In this manner, these clusters concentrate Nck, N-WASP and the ARP2/3 complex thereby enhancing actin filament assembly (Su et al., 2016). In addition, zona occludens proteins were shown to self-organize at tight junctions by phase separation and attract and concentrate cytoskeletal adaptor proteins and F-actin (Beutel et al., 2019). Together, this shows that phase separation at the plasma membrane creates membraneless compartments that influence the local actin cytoskeleton by enhancing actin polymerization. It is conceivable that other membrane proteins can drive the formation of phase-separated clusters and future research will have to determine how widespread this mechanism is. For example, it is interesting to speculate that this may be involved in axon guidance since extracellular cues are known to induce local changes in the actin cytoskeleton, thereby influencing growth cone steering (Dorskind and Kolodkin, 2020).

Microtubule nucleation can also be driven by phase separation. Several recent studies have shown that phase separation may be an important mechanism during cell division by promoting microtubule nucleation (Ong and Torres, 2020). *In vitro* work using *C. elegans* proteins revealed that tubulin concentration was increased approximately 4-fold by the formation of biomolecular condensates at the centrosome containing the scaffold protein SPD-5, which in turn recruits the microtubule effector protein homologs of XMAP215/CKAP5 and TPX2. This increase in tubulin concentration was sufficient to drive microtubule nucleation (Figure 2D; Woodruff et al., 2017). The microtubule-associated protein PLK4 was also shown to self-assemble into condensates and recruit and concentrate tubulin thereby promoting the formation of a microtubule organizing center (Montenegro Gouveia et al., 2018). In a similar manner, several other proteins have been shown to promote microtubule assembly by concentrating tubulin via phase separation at mitotic spindles (Jiang et al., 2015; So et al., 2019; King and Petry, 2020; Safari et al., 2020). In addition, it was shown that tubulin can be concentrated into liquid-like phase separated droplets *in vitro* formed by the microtubule-associated protein Tau (Hernandez-Vega et al., 2017). This concentrated tubulin polymerizes within these droplets and forms microtubule bundles (Figure 2D). Interestingly, tau droplets have been observed in neurons (Wegmann et al., 2018). This mechanism provides an interesting possibility for the formation of local, non-centrosomal microtubule nucleation (Kuijpers and Hoogenraad, 2011).

Finally, it has been suggested that the actin cytoskeleton itself can exhibit liquid-like behavior and form condensates (Weirich et al., 2017). In the presence of the crosslinker filamin, actin filaments can be condensed into a liquid droplet phase. This enables a mechanism to control the morphology and dynamics of the actin cytoskeleton.

Together, these recent studies provide compelling evidence that phase separation and the formation of condensates can promote the nucleation and remodeling of the cytoskeleton at specific subcellular locations.

## Interactions Between Membrane-Bound Organelles and Biomolecular Condensates in Conjunction With the Cytoskeleton

As discussed in the previous two sections, it has become clear that both membrane-bound organelles and biomolecular condensates are directly or indirectly contacting the cytoskeleton. Increasing evidence shows that they also frequently form contacts with each other, often in conjunction with the cytoskeleton (Salogiannis and Reck-Peterson, 2017; Bethune et al., 2019; Zhao and Zhang, 2020). The functional roles of these contacts and their interplay with the cytoskeleton are just starting to emerge and recent advances will be discussed in this section.

### Biomolecular Condensates Can Associate With Membrane-Bound Organelles

One of the first examples of an association between biomolecular condensates and membrane-bound organelles was shown when GW-bodies were found to associate with LAMP1- and CD63-positive lysosomes/multivesicular bodies (MVBs) (Gibbings et al., 2009; Lee et al., 2009). These GW-bodies, containing the miRNA processing proteins GW-182 and Ago2, are closely related to P-bodies and share several components but likely represent different condensates (Patel et al., 2016). The formation of MVBs was found to be important for miRNA-mediated gene silencing (Gibbings et al., 2009; Lee et al., 2009).

Huang et al. revealed that a large proportion of P-bodies can interact with mitochondria in various cell lines (Huang et al., 2011). Electron microscopy and live cell imaging analysis in HeLa cells showed that P-bodies can form close and dynamic contacts with mitochondria. The authors state they did not observe a close association of P-bodies with other organelles, such as the ER, endosomes or vacuoles, with electron microscopy. Depolymerization of the microtubule network by vinblastine treatment increased the number of P-bodies but decreased the association frequency with mitochondria, suggesting an intact microtubule network is required for these contacts. P-body depletion did not influence mitochondrial morphology and function and CCCP-induced mitochondrial uncoupling did not affect the size or number of P-bodies or their contact with mitochondria. By contrast, CCCP treatment drastically affected miRNA- and siRNA-mediated gene silencing, possibly by decreasing the accumulation of Ago2 in P-bodies (Huang et al., 2011).

In addition to mitochondria, a clear association between the ER and P-bodies has been established. In yeast, P-bodies only form under stress conditions and in this model system they were observed in close proximity to the ER with both co-localization analysis using fluorescence microscopy and by electron microscopy imaging (Kilchert et al., 2010). Differential centrifugation experiments to isolate ER membranes confirmed that P-bodies are physically associated to the ER (Kilchert et al., 2010; Weidner et al., 2014). Subsequently, the yeast ER proteins Scp160 and Brf1 (closest human homologs are vigilin and FMRP) were found to interact with several P-body components but deletion of these proteins did not impair P-body localization to the ER (Weidner et al., 2014). Although an association with the ER was not observed in HeLa cells (Huang et al., 2011), a very recent and elegant study used live-cell imaging methods to reveal that P-bodies, as well as stress granules can indeed form dynamic contacts with the ER in U2OS cells (Lee et al., 2020). The authors also investigated the relationship between ER shape and ER translational status with P-body formation and found that ER tubules and inhibition of translation promote P-body formation. Intriguingly, they revealed that fission of both P-bodies and stress granules occurs at ER contact sites (Lee et al., 2020). Two other recent studies also described associations of RNP or stress granules with the ER. In *Xenopus* RGC axons, a close association of RNA granules was observed with the ER (Cioni et al., 2019) and in U2OS cells a high colocalization of G3BP1-positive stress granules with the ER was found (Liao et al., 2019). The functional relevance of contacts between the ER and P-bodies or other RNP granules remains to be determined but possibly includes the exchange of mRNAs and regulation of RNP granule size. Although RNP granules do not contain a membrane, it is conceivable that the microtubule cytoskeleton is involved in RNP granule fission events analogous to the regulation of fission of membrane-bound organelles by the ER (Friedman et al., 2010; Rowland et al., 2014). In addition, since both P-bodies and stress granules interact with the ER and they all associate with microtubules, it is possible that the ER plays a role in the docking of P-bodies to stress granules. Moreover, it is unknown if the interaction of P-bodies and stress granules with the ER can reciprocally influence ER organization.

Two additional types of RNP granules have been shown to be associated with the ER; TIS granules and Sec bodies. TIS granules, containing the RBPs TIS11B and HuR as well as the chaperone protein HSPA8, are intertwined with ER sheets and concentrate specific mRNAs with AU-rich elements (Ma and Mayr, 2018). These gel-like granules are present under physiological conditions and have a tubular mesh-like morphology, driven by RNA-RNA interactions, that is different from the spherical morphology described for other RNP granules (Ma et al., 2020). The formation of TIS granules and its association to ER sheets enables the local translation of specific AU-rich mRNAs at the ER.

Sec bodies represent another stress-induced condensate and are also related to the ER since they are formed at ER exit sites (ERES) upon amino acid starvation in *Drosophila* S2 cells (Zacharogianni et al., 2014; Aguilera-Gomez et al., 2016). These condensates protect ERES components during stress and act as a reservoir to reinitiate secretory vesicle formation after stress relief. Thus far, it remains unknown if these Sec bodies also exist in mammalian cells and if their formation and disassembly require an intact cytoskeleton.

Together, these studies illustrate that interactions between biomolecular condensates and membrane-bound organelles occur frequently, but for most of these interactions the exact mechanisms and functions as well as their relationship with the cytoskeleton will require further investigation.

### Biomolecular Condensates Can “Hitchhike” on Membrane-Bound Organelles for Transport

In recent years, it has become clear that both RNP transport granules and stress granules can also be transported via the cytoskeleton by “hitchhiking” on membrane-bound organelles. During asymmetric cell division in budding yeast, the cortical ER, a tubular ER structure which is in close contact with the plasma membrane, is transported by myosin motor proteins on the actin cytoskeleton to the daughter cell (Estrada et al., 2003). During this process, it has been shown that several mRNAs can be co-transported on the ER membrane via the adaptor RBP She2p to the yeast bud for local translation (Schmid et al., 2006; Aronov et al., 2007). This represents a first example of coordinated co-transport of biomolecular condensates (RNPs) with membrane-bound organelles on the cytoskeleton. Another clear and one of the best-studied examples of “hitchhiking” of RNP granules with membrane-bound organelles was found during the highly polarized growth of hyphae of the fungus *Ustilago Maydis*. In this organism, transport of several mRNAs by the RNA-binding protein Rrm4 is essential for this polarized growth (Konig et al., 2009; Bethune et al., 2019). It was found that Rrm4 colocalizes and co-moves only with motile but not static Rab5a-positive early endosomes that travel along MTs driven by kinesin-3 or dynein motors (Baumann et al., 2012, 2014). Several specific mRNAs, including mRNAs coding for all four septins (*cdc3, cdc10, cdc11*, and *cdc12*), co-transport together with ribosomes on these endosomes. These septin proteins can form heteromeric complexes on the cytoplasmic surface of endosomes in a Rrm4-dependent matter, indicating local translation occurs on these endosomes (Baumann et al., 2014; Higuchi et al., 2014). Since these findings, many components of this transport system in fungal hyphae, including adaptor proteins and additional mRNAs and RBPs have been identified (Pohlmann et al., 2015; Jankowski et al., 2019; Olgeiser et al., 2019).

In neurons, a similar hitchhiking mechanism involving RNP granules and endosomes was recently uncovered. Live-cell imaging of *Xenopus Laevis* RGC axons revealed that RNP granules often associate and co-transport with both Rab5-positive early endosomes and Rab7-positive late endosomes (Cioni et al., 2019). Another study found frequent associations and co-movement of heat shock-induced stress granules with LAMP1-positive lysosomes in a microtubule and motor-dependent fashion in U2OS cells and iPSC-derived cortical neurons (Liao et al., 2019). Interestingly, the authors utilized APEX2-mediated proximity labeling analysis in human iPSC-induced neurons to identify that Annexin 11 (ANXA11), a RNA granule–associated phosphoinositide binding protein also found in stress granules (Markmiller et al., 2018), tethers stress granules to actively transported lysosomes. Interestingly, stress granule-lysosome contacts were observed at the ER in U2OS cells (Liao et al., 2019). As described above, we recently found that ER-lysosome contacts in the soma of neurons are important for the axonal translocation of lysosomes (Özkan et al., 2020). Together, this raises the interesting possibility that neuronal somatic ER tubules provide a platform for mRNA localization or transfer to lysosomes and likely indirectly regulate RNP transport into axons by influencing lysosome distribution.

RNP granule hitchhiking provides a mechanism for neurons to localize mRNAs into axons for their local translation and indeed, the axonal translation of two mitochondrial mRNAs (*laminb2* and *vdac*) was shown to occur on Rab7a-positive late endosomes (Cioni et al., 2019). Interestingly, late endosomes that colocalized with RNA granules and stained positive for puromycin (indicating protein synthesis), were often observed in close proximity to mitochondria, suggesting newly synthesized proteins may be delivered to mitochondria by late endosomes.

An important role for endosomes in mRNA localization and translation is further supported by a recent study that showed both translation-dependent and -independent association of various mRNAs to early endosomes in HeLa cells (Popovic et al., 2020). This study provided important insights into which mRNAs localize to early endosomes and it will be interesting to determine if endosome-localized mRNAs are conserved across cell types and organisms or if there are cell-type specific or even context-dependent differences in endosomal mRNA localization and translation. In addition, future research will have to clarify why there seem to be cell-type specific differences in which type of endosome (early, late or lysosomes) is mainly responsible for mRNA transport and localized mRNA translation (Baumann et al., 2014; Cioni et al., 2019; Liao et al., 2019; Popovic et al., 2020).

Interestingly, two recent reports described a role for late endosomes/lysosomes in the transport of microRNAs in axons. Gershoni-Emek et al. (2018) showed that miRNAs and RNAi proteins colocalized and co-moved with acidic compartments marked by Lysotracker and frequently dock at mitochondria. The second report convincingly showed that pre-miRNAs are transported on CD63-positive late endosomes/lysosomes in RGC axons (Corradi et al., 2020). Since RNAi proteins and possibly miRNAs are known to reside in P-bodies or GW-bodies, these studies raise the interesting possibility that P-bodies or GW-bodies also utilize hitchhiking on endo-lysosomes for their co-transport. This possibility is supported by the aforementioned association of CD63- or LAMP1-positive multivesicular bodies/lysosomes with GW-bodies in *Drosophila* and human cells (Gibbings et al., 2009; Lee et al., 2009).

Finally, RNP granule hitchhiking on endosomal compartments can influence the local cytoskeleton by localizing and translating certain cytoskeletal mRNAs at specific subcellular locations. As mentioned above, all four mRNAs encoding the cytoskeletal septin proteins are localized to early endosomes in fungal hyphae and their translation on these endosomes is essential for correct septin filamentation and fungal growth (Baumann et al., 2014). In addition, β-actin mRNA was found to co-move on late endosomes/lysosomes in axons and several mRNAs coding for cytoskeletal proteins were identified on EEA1-positive early endosomes in HeLa cells (Cioni et al., 2019; Liao et al., 2019; Popovic et al., 2020).

Together, these studies show that there is an intricate interplay between (multiple) membrane-bound organelles, biomolecular condensates and the cytoskeleton which is crucial for maintaining the proper distribution and function of both the organelles themselves as well as the cytoskeleton.

## Concluding Remarks and Future Perspectives

It has long been clear that the distinct domains that organize a cell and allow the separation of cellular/biochemical processes, such as membrane-bound organelles, biomolecular condensates, and the cytoskeleton, do not merely perform their functions by themselves. Rather, they have to be well-coordinated by communicating with each other through frequent and dynamic interactions. The extent of complexity of these interactions is only recently becoming clear and has greatly advanced with the advent of new imaging and biochemical techniques. For example, the recent development of multispectral imaging approaches allowed the simultaneous imaging of six different membrane-bound organelles and revealed a complex organelle interactome that depends on an intact microtubule cytoskeleton (Valm et al., 2017). In addition, super-resolution imaging approaches that enable live-cell multi-color imaging at a high spatiotemporal resolution such as the above discussed GI-SIM imaging (Guo et al., 2018) revealed new types of inter-organelle interactions in conjunction with the cytoskeleton and their effect on organelle dynamics. These imaging techniques can be further applied in the future to study the dynamics of interactions between the cytoskeleton, (multiple) membrane-bound organelles and biomolecular condensates in different cell types and different cellular states and conditions. Moreover, the recent and continuing development of novel split reporter systems is improving the visualization of inter-organelle contacts (reviewed by Scorrano et al., 2019) and novel biochemical approaches such as proximity labeling will advance our understanding of the molecular makeup of each cellular compartment. The latter is especially important because it is often unknown which proteins mediate and regulate these contacts and these novel techniques will be able to identify possible tethering and regulatory proteins (Lam et al., 2015; Fazal et al., 2019; Gingras et al., 2019).

**FIGURE 3:**
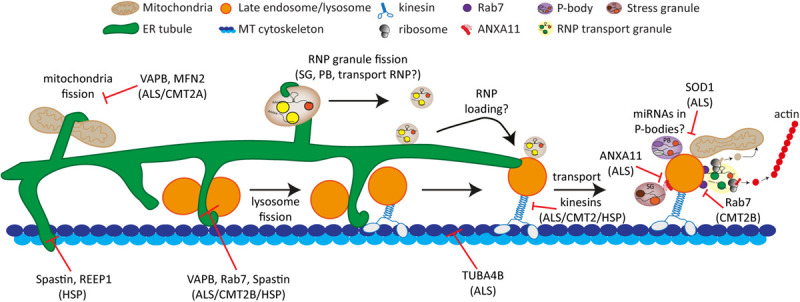
Interplay between membrane-bound organelles, biomolecular condensates, and the cytoskeleton. A model depicting the complex interactions between membrane-bound organelles, biomolecular condensates and the cytoskeleton. Membrane-bound organelles (ER, mitochondria, endo-lysosomes) form contacts with each other and the microtubule cytoskeleton and can also form dynamic contacts with biomolecular condensates. For simplicity, transport granules, P-bodies and stress granules are depicted collectively as “RNP granules” for granule fission. These interactions and their complex interplay regulate the organization and remodeling of membrane-bound organelles, biomolecular condensates and the cytoskeleton. Mutations in various proteins influencing these complex interactions are linked to several neurodegenerative diseases and are indicated in the model.

The highly complex interactions and interplay illustrated in this review may be especially important for the correct organization and functioning of morphologically complex and highly polarized cells such as neurons. Many studies have shown that dysfunction of the cytoskeleton, several membrane-bound organelles and biomolecular condensates is implicated in neuron dysfunction and neurological disorders (Figure 3; Fowler et al., 2019; Sleigh et al., 2019; Zbinden et al., 2020). However, the role of the complex interactions and interplay between them in disease pathogenesis is much less clear. Several lines of evidence support the notion that disruptions of this complex interplay may be an important causative factor in neurological disorders. For instance, mutations in ANXA11, the protein that was found to tether stress granules to lysosomes for their hitchhiking-based transport, cause ALS. These ALS-associated mutations in ANXA11 impair its tethering function and reduce RNA transport into the axon (Figure 3; Liao et al., 2019). Mutations in the late endosome/lysosome protein Rab7 that cause the neuropathy Charcot-Marie-Tooth Type 2B impair the axonal synthesis of mitochondrial proteins and led to reduced mitochondrial function and axonal viability (Figure 3). Although the exact mechanism leading to this phenotype is not entirely clear, it is conceivable that this is a consequence of impaired local translation of mitochondrial mRNAs on Rab7-positive endosomes that would normally be delivered to nearby mitochondria via the cytoskeleton to sustain their function (Cioni et al., 2019). In addition, the lysosome-associated axonal transport of a miRNA and RNAi proteins, possibly contained in P-bodies (Gibbings et al., 2009; Lee et al., 2009), and their docking at mitochondria was altered in ALS-associated SOD1 mutant neurons (Figure 3; Gershoni-Emek et al., 2018). Finally, it is known that mutations in the ER-resident and MT-severing protein Spastin cause the neurodegenerative disease HSP (Blackstone, 2012). As mentioned above, Spastin interacts with IST1 at ER-late endosome/lysosome contact sites to promote endosomal fission (Figure 3; Allison et al., 2013). In both primary neurons from Spastin-HSP mice and HSP-patient-derived iPSC neurons enlarged lysosomes were observed, suggesting a disturbance of the complex interaction between the ER, late endosome/lysosomes and MTs although the exact mechanism that leads to the enlarged lysosomes in HSP patients remains to be determined (Allison et al., 2017).

In addition, several exciting recent studies have shown that there is crosstalk between cytoskeletal components. These components are inter-linked by proteins that directly or indirectly bind to the MT and actin cytoskeleton, and this crosstalk has been shown to play essential roles in cell division, migration, immune synapse and axonal growth (Rodriguez et al., 2003; Mohan and John, 2015; van de Willige et al., 2019; Biswas and Kalil, 2018; Martin-Cofreces and Sanchez-Madrid, 2018). The role of this cytoskeletal crosstalk on organelle dynamics remains largely unexplored.

The recent technical advances mentioned above open up the possibility to further investigate these complex interactions and study the consequence of disease mutations, which will likely increase our knowledge on disease pathogenesis.

## Author Contributions

MK and NÖ conceived and wrote the manuscript and designed the figures. GF contributed to manuscript writing, provided feedback, and proofread the manuscript. All authors read and approved the manuscript before submission.

## Conflict of Interest

The authors declare that the research was conducted in the absence of any commercial or financial relationships that could be construed as a potential conflict of interest.

## References

[B1] Abrisch R. G. Gumbin S. C. Wisniewski B. T. Lackner L. L. Voeltz G. K. (2020). Fission and fusion machineries converge at ER contact sites to regulate mitochondrial morphology. *J. Cell Biol.* 219:e201911122. 10.1083/jcb.201911122 32328629 PMC7147108

[B2] Aguilera-Gomez A. Van Oorschot M. M. Veenendaal T. Rabouille C. (2016). In vivo vizualisation of mono-ADP-ribosylation by dPARP16 upon amino-acid starvation. *eLife* 5:e21475. 10.7554/eLife.21475 PMC5127640 27874829

[B3] Aizer A. Brody Y. Ler L. W. Sonenberg N. Singer R. H. Shav-Tal Y. (2008). The dynamics of mammalian P body transport, assembly, and disassembly in vivo. *Mol. Biol. Cell.* 19 4154–4166. 10.1091/mbc.e08-05-0513 18653466 PMC2555939

[B4] Akhmanova A. Steinmetz M. O. (2019). Microtubule minus-end regulation at a glance. *J. Cell. Sci.* 132:jcs227850. 10.1242/jcs.227850 31175152

[B5] Alberti S. Dormann D. (2019). Liquid-liquid phase separation in disease. *Annu. Rev. Genet.* 53 171–194. 10.1146/annurev-genet-112618-043527 31430179

[B6] Allison R. Edgar J. R. Pearson G. Rizo T. Newton T. Gunther S. (2017). Defects in Er-endosome contacts impact lysosome function in hereditary spastic paraplegia. *J. Cell Biol.* 216 1337–1355. 10.1083/jcb.201609033 28389476 PMC5412567

[B7] Allison R. Lumb J. H. Fassier C. Connell J. W. Ten Martin D. Seaman M. N. (2013). An ESCRT-spastin interaction promotes fission of recycling tubules from the endosome. *J. Cell Biol.* 202 527–543. 10.1083/jcb.201211045 23897888 PMC3734076

[B8] Aronov S. Gelin-Licht R. Zipor G. Haim L. Safran E. Gerst J. E. (2007). mRNAs encoding polarity and exocytosis factors are cotransported with the cortical endoplasmic reticulum to the incipient bud in *Saccharomyces cerevisiae*. *Mol. Cell. Biol.* 27 3441–3455. 10.1128/mcb.01643-06 17339339 PMC1899969

[B9] Aulas A. Fay M. M. Lyons S. M. Achorn C. A. Kedersha N. Anderson P. (2017). Stress-specific differences in assembly and composition of stress granules and related foci. *J. Cell. Sci.* 130 927–937. 10.1242/jcs.199240 28096475 PMC5358336

[B10] Ayache J. Benard M. Ernoult-Lange M. Minshall N. Standart N. Kress M. (2015). P-body assembly requires DDX6 repression complexes rather than decay or Ataxin2/2L complexes. *Mol. Biol. Cell.* 26 2579–2595. 10.1091/mbc.e15-03-0136 25995375 PMC4501357

[B11] Banani S. F. Lee H. O. Hyman A. A. Rosen M. K. (2017). Biomolecular condensates: organizers of cellular biochemistry. *Nat. Rev. Mol. Cell. Biol.* 18 285–298. 10.1038/nrm.2017.7 28225081 PMC7434221

[B12] Banjade S. Rosen M. K. (2014). Phase transitions of multivalent proteins can promote clustering of membrane receptors. *eLife* 3:e04123. 10.7554/eLife.04123 PMC4238058 25321392

[B13] Baumann S. Komissarov A. Gili M. Ruprecht V. Wieser S. Maurer S. P. (2020). A reconstituted mammalian Apc-kinesin complex selectively transports defined packages of axonal mRNAs. *Sci. Adv.* 6:eaaz1588. 10.1126/sciadv.aaz1588 32201729 PMC7069705

[B14] Baumann S. Konig J. Koepke J. Feldbrugge M. (2014). Endosomal transport of septin mrna and protein indicates local translation on endosomes and is required for correct septin filamentation. *EMBO Rep.* 15 94–102. 10.1002/embr.201338037 24355572 PMC4303453

[B15] Baumann S. Pohlmann T. Jungbluth M. Brachmann A. Feldbrugge M. (2012). Kinesin-3 and dynein mediate microtubule-dependent co-transport of mRNPs and endosomes. *J. Cell. Sci.* 125 2740–2752. 10.1242/jcs.101212 22357951

[B16] Bethune J. Jansen R. P. Feldbrugge M. Zarnack K. (2019). Membrane-associated RNA-binding proteins orchestrate organelle-coupled translation. *Trends Cell Biol.* 29 178–188. 10.1016/j.tcb.2018.10.005 30455121

[B17] Beutel O. Maraspini R. Pombo-Garcia K. Martin-Lemaitre C. Honigmann A. (2019). Phase separation of zonula occludens proteins drives formation of tight junctions. *Cell* 179 923.e11–936.e11. 31675499 10.1016/j.cell.2019.10.011

[B18] Biswas S. Kalil K. (2018). The microtubule-associated protein tau mediates the organization of microtubules and their dynamic exploration of actin-rich lamellipodia and filopodia of cortical growth cones. *J. Neurosci.* 38 291–307. 10.1523/jneurosci.2281-17.2017 29167405 PMC5761611

[B19] Blackstone C. (2012). Cellular pathways of hereditary spastic paraplegia. *Annu. Rev. Neurosci.* 35 25–47. 10.1146/annurev-neuro-062111-150400 22540978 PMC5584684

[B20] Boeynaems S. Alberti S. Fawzi N. L. Mittag T. Polymenidou M. Rousseau F. (2018). Protein phase separation: a new phase in cell biology. *Trends Cell Biol.* 28 420–435. 10.1016/j.tcb.2018.02.004 29602697 PMC6034118

[B21] Bonifacino J. S. Neefjes J. (2017). Moving and positioning the endolysosomal system. *Curr. Opin. Cell Biol.* 47 1–8. 10.1016/j.ceb.2017.01.008 28231489 PMC5537022

[B22] Braakman I. Hebert D. N. (2013). Protein folding in the endoplasmic reticulum. *Cold Spring Harb. Perspect. Biol.* 5:a013201. 10.1101/cshperspect.a013201 PMC3632058 23637286

[B23] Brengues M. Teixeira D. Parker R. (2005). Movement of eukaryotic mrnas between polysomes and cytoplasmic processing bodies. *Science* 310 486–489. 10.1126/science.1115791 16141371 PMC1863069

[B24] Buchan J. R. (2014). mRNP granules. Assembly, function, and connections with disease. *RNA Biol.* 11 1019–1030. 10.4161/15476286.2014.972208 25531407 PMC4615263

[B25] Burute M. Kapitein L. C. (2019). Cellular logistics: unraveling the interplay between microtubule organization and intracellular transport. *Annu. Rev. Cell. Dev. Biol.* 35 29–54. 10.1146/annurev-cellbio-100818-125149 31394046

[B26] Buxbaum A. R. Haimovich G. Singer R. H. (2015). In the right place at the right time: visualizing and understanding mRNA localization. *Nat. Rev. Mol. Cell Biol.* 16 95–109. 10.1038/nrm3918 25549890 PMC4484810

[B27] Carbonaro M. O’brate A. Giannakakou P. (2011). Microtubule disruption targets HIF-1alpha mRNA to cytoplasmic P-bodies for translational repression. *J. Cell Biol.* 192 83–99. 10.1083/jcb.201004145 21220510 PMC3019555

[B28] Case L. B. Zhang X. Ditlev J. A. Rosen M. K. (2019). Stoichiometry controls activity of phase-separated clusters of actin signaling proteins. *Science* 363 1093–1097. 10.1126/science.aau6313 30846599 PMC6784323

[B29] Chada S. R. Hollenbeck P. J. (2004). Nerve growth factor signaling regulates motility and docking of axonal mitochondria. *Curr. Biol.* 14 1272–1276. 10.1016/j.cub.2004.07.027 15268858

[B30] Chakrabarti R. Ji W. K. Stan R. V. De juan sanz J. Ryan T. A. Higgs H. N. (2018). Inf2-mediated actin polymerization at the ER stimulates mitochondrial calcium uptake, inner membrane constriction, and division. *J. Cell. Biol.* 217 251–268. 10.1083/jcb.201709111 29142021 PMC5748994

[B31] Chang W. Zaarour R. F. Reck-Peterson S. Rinn J. Singer R. H. Snyder M. (2008). Myo2p, a class V myosin in budding yeast, associates with a large ribonucleic acid-protein complex that contains mRNAs and subunits of the RNA-processing body. *RNA* 14 491–502. 10.1261/rna.665008 18218704 PMC2248268

[B32] Chen J. V. Buchwalter R. A. Kao L. R. Megraw T. L. (2017). A splice variant of centrosomin converts mitochondria to microtubule-organizing centers. *Curr. Biol.* 27 1928.e6–1940.e6. 28669756 10.1016/j.cub.2017.05.090 PMC6147254

[B33] Chen S. Novick P. Ferro-Novick S. (2013). ER structure and function. *Curr. Opin. Cell. Biol.* 25 428–433. 23478217 10.1016/j.ceb.2013.02.006 PMC5614462

[B34] Chen Y. Sheng Z. H. (2013). Kinesin-1-syntaphilin coupling mediates activity-dependent regulation of axonal mitochondrial transport. *J. Cell Biol.* 202 351–364. 10.1083/jcb.201302040 23857772 PMC3718985

[B35] Chernov K. G. Barbet A. Hamon L. Ovchinnikov L. P. Curmi P. A. Pastre D. (2009). Role of microtubules in stress granule assembly: microtubule dynamical instability favors the formation of micrometric stress granules in cells. *J. Biol. Chem.* 284 36569–36580. 10.1074/jbc.m109.042879 19843517 PMC2794772

[B36] Cioni J. M. Koppers M. Holt C. E. (2018). Molecular control of local translation in axon development and maintenance. *Curr. Opin. Neurobiol.* 51 86–94. 10.1016/j.conb.2018.02.025 29549711

[B37] Cioni J. M. Lin J. Q. Holtermann A. V. Koppers M. Jakobs M. A. H. Azizi A. (2019). Late endosomes act as mrna translation platforms and sustain mitochondria in axons. *Cell* 176 56.e15–72.e15. 30612743 10.1016/j.cell.2018.11.030 PMC6333918

[B38] Coldwell M. J. Cowan J. L. Vlasak M. Mead A. Willett M. Perry L. S. (2013). Phosphorylation of eIF4GII and 4E-BP1 in response to nocodazole treatment: a reappraisal of translation initiation during mitosis. *Cell Cycle* 12 3615–3628. 10.4161/cc.26588 24091728 PMC3903713

[B39] Corradi E. Dalla Costa I. Gavoci A. Iyer A. Roccuzzo M. Otto T. A. (2020). Axonal precursor miRNAs hitchhike on endosomes and locally regulate the development of neural circuits. *EMBO J.* 39:e102513. 10.15252/embj.2019102513 PMC7073465 32073171

[B40] Cougot N. Bhattacharyya S. N. Tapia-Arancibia L. Bordonne R. Filipowicz W. Bertrand E. (2008). Dendrites of mammalian neurons contain specialized P-body-like structures that respond to neuronal activation. *J. Neurosci.* 28 13793–13804. 10.1523/jneurosci.4155-08.2008 19091970 PMC6671906

[B41] Das S. Singer R. H. Yoon Y. J. (2019). The travels of mRNAs in neurons: do they know where they are going? *Curr. Opin. Neurobiol.* 57 110–116. 10.1016/j.conb.2019.01.016 30784978 PMC6650148

[B42] Decker C. J. Parker R. (2012). P-bodies and stress granules: possible roles in the control of translation and mRNA degradation. *Cold Spring Harb. Perspect. Biol.* 4:a012286. 10.1101/cshperspect.a012286 22763747 PMC3428773

[B43] Dekker-Ohno K. Hayasaka S. Takagishi Y. Oda S. Wakasugi N. Mikoshiba K. (1996). Endoplasmic reticulum is missing in dendritic spines of Purkinje cells of the ataxic mutant rat. *Brain Res.* 714 226–230. 10.1016/0006-8993(95)01560-4 8861629

[B44] Dorskind J. M. Kolodkin A. L. (2020). Revisiting and refining roles of neural guidance cues in circuit assembly. *Curr. Opin. Neurobiol.* 66 10–21. 10.1016/j.conb.2020.07.005 32823181 PMC10725571

[B45] Dreier L. Rapoport T. A. (2000). In vitro formation of the endoplasmic reticulum occurs independently of microtubules by a controlled fusion reaction. *J. Cell. Biol.* 148 883–898. 10.1083/jcb.148.5.883 10704440 PMC2174540

[B46] Edelmann F. T. Schlundt A. Heym R. G. Jenner A. Niedner-Boblenz A. Syed M. I. (2017). Molecular architecture and dynamics of Ash1 mrna recognition by its mrna-transport complex. *Nat. Struct. Mol. Biol.* 24 152–161. 10.1038/nsmb.3351 28092367

[B47] English A. R. Zurek N. Voeltz G. K. (2009). Peripheral ER structure and function. *Curr. Opin. Cell. Biol.* 21 596–602. 10.1016/j.ceb.2009.04.004 19447593 PMC2753178

[B48] Estrada P. Kim J. Coleman J. Walker L. Dunn B. Takizawa P. (2003). Myo4p and She3p are required for cortical ER inheritance in *Saccharomyces cerevisiae*. *J. Cell. Biol.* 163 1255–1266. 10.1083/jcb.200304030 14691136 PMC2173705

[B49] Etienne-Manneville S. (2018). Cytoplasmic intermediate filaments in cell biology. *Annu. Rev. Cell. Dev. Biol.* 34 1–28. 10.1146/annurev-cellbio-100617-062534 30059630

[B50] Eystathioy T. Chan E. K. Tenenbaum S. A. Keene J. D. Griffith K. Fritzler M. J. (2002). A phosphorylated cytoplasmic autoantigen, GW182, associates with a unique population of human mRNAs within novel cytoplasmic speckles. *Mol. Biol. Cell.* 13 1338–1351. 10.1091/mbc.01-11-0544 11950943 PMC102273

[B51] Fagone P. Jackowski S. (2009). Membrane phospholipid synthesis and endoplasmic reticulum function. *J. Lipid Res.* 50(Suppl.), S311–S316. 18952570 10.1194/jlr.R800049-JLR200 PMC2674712

[B52] Farias G. G. Freal A. Tortosa E. Stucchi R. Pan X. Portegies S. (2019). Feedback-driven mechanisms between microtubules and the endoplasmic reticulum instruct neuronal polarity. *Neuron* 102 184.e8–201.e8. 30772082 10.1016/j.neuron.2019.01.030

[B53] Fazal F. M. Han S. Parker K. R. Kaewsapsak P. Xu J. Boettiger A. N. (2019). Atlas of Subcellular RNA localization revealed by APEX-Seq. *Cell* 178 473.e26–490.e26. 31230715 10.1016/j.cell.2019.05.027 PMC6786773

[B54] Fonseca T. B. Sanchez-Guerrero A. Milosevic I. Raimundo N. (2019). Mitochondrial fission requires Drp1 but not dynamins. *Nature* 570 E34–E42. 31217603 10.1038/s41586-019-1296-y

[B55] Formicola N. Vijayakumar J. Besse F. (2019). Neuronal ribonucleoprotein granules: dynamic sensors of localized signals. *Traffic* 20 639–649. 31206920 10.1111/tra.12672

[B56] Fowler P. C. Garcia-Pardo M. E. Simpson J. C. O’sullivan N. C. (2019). Neurodegeneration: the central role for ER contacts in neuronal function and axonopathy, lessons from hereditary spastic paraplegias and related diseases. *Front. Neurosci.* 13:1051. 10.3389/fnins.2019.01051 31680803 PMC6801308

[B57] Friedman J. R. Dibenedetto J. R. West M. Rowland A. A. Voeltz G. K. (2013). Endoplasmic reticulum-endosome contact increases as endosomes traffic and mature. *Mol. Biol. Cell.* 24 1030–1040. 10.1091/mbc.e12-10-0733 23389631 PMC3608491

[B58] Friedman J. R. Lackner L. L. West M. Dibenedetto J. R. Nunnari J. Voeltz G. K. (2011). Er tubules mark sites of mitochondrial division. *Science* 334 358–362. 10.1126/science.1207385 21885730 PMC3366560

[B59] Friedman J. R. Voeltz G. K. (2011). The ER in 3D: a multifunctional dynamic membrane network. *Trends Cell Biol.* 21 709–717. 10.1016/j.tcb.2011.07.004 21900009 PMC3221873

[B60] Friedman J. R. Webster B. M. Mastronarde D. N. Verhey K. J. Voeltz G. K. (2010). ER sliding dynamics and Er-mitochondrial contacts occur on acetylated microtubules. *J. Cell. Biol.* 190 363–375. 10.1083/jcb.200911024 20696706 PMC2922647

[B61] Fu M. M. Mcalear T. S. Nguyen H. Oses-Prieto J. A. Valenzuela A. Shi R. D. (2019). The golgi outpost protein TPPP nucleates microtubules and is critical for myelination. *Cell* 179 132.e14–146.e14. 31522887 10.1016/j.cell.2019.08.025 PMC7214773

[B62] Fujimura K. Katahira J. Kano F. Yoneda Y. Murata M. (2009). Microscopic dissection of the process of stress granule assembly. *Biochim. Biophys. Acta* 1793 1728–1737. 10.1016/j.bbamcr.2009.08.010 19733198

[B63] Fukuda Y. Pazyra-Murphy M. F. Tasdemir-Yilmaz O. E. Li Y. Rose L. Yeoh Z. C. (2020). Fast transport of RNA granules by direct interactions with KIF5A/KLC1 motors prevents axon degeneration. *BioRxiv* [Preprint]. 10.1101/2020.02.02.931204

[B64] Garmendia-Torres C. Skupin A. Michael S. A. Ruusuvuori P. Kuwada N. J. Falconnet D. (2014). Unidirectional P-body transport during the yeast cell cycle. *PLoS One* 9:e99428. 10.1371/journal.pone.0099428 24918601 PMC4053424

[B65] Gavin A. C. Aloy P. Grandi P. Krause R. Boesche M. Marzioch M. (2006). Proteome survey reveals modularity of the yeast cell machinery. *Nature* 440 631–636. 10.1038/nature04532 16429126

[B66] Geitmann A. Nebenfuhr A. (2015). Navigating the plant cell: intracellular transport logistics in the green kingdom. *Mol. Biol. Cell.* 26 3373–3378. 10.1091/mbc.e14-10-1482 26416952 PMC4591683

[B67] Gershoni-Emek N. Altman T. Ionescu A. Costa C. J. Gradus-Pery T. Willis D. E. (2018). Localization of RNAi machinery to axonal branch points and growth cones is facilitated by mitochondria and is disrupted in Als. *Front. Mol. Neurosci.* 11:311. 10.3389/fnmol.2018.00311 30233312 PMC6134038

[B68] Gibbings D. J. Ciaudo C. Erhardt M. Voinnet O. (2009). Multivesicular bodies associate with components of mirna effector complexes and modulate miRNA activity. *Nat. Cell. Biol.* 11 1143–1149. 10.1038/ncb1929 19684575

[B69] Gingras A. C. Abe K. T. Raught B. (2019). Getting to know the neighborhood: using proximity-dependent biotinylation to characterize protein complexes and map organelles. *Curr. Opin. Chem. Biol.* 48 44–54. 10.1016/j.cbpa.2018.10.017 30458335

[B70] Gopal P. P. Nirschl J. J. Klinman E. Holzbaur E. L. (2017). Amyotrophic lateral sclerosis-linked mutations increase the viscosity of liquid-like TDP-43 RNP granules in neurons. *Proc. Natl. Acad. Sci. U.S.A.* 114 E2466–E2475. 28265061 10.1073/pnas.1614462114 PMC5373408

[B71] Goyal U. Blackstone C. (2013). Untangling the web: mechanisms underlying ER network formation. *Biochim. Biophys. Acta* 1833 2492–2498. 10.1016/j.bbamcr.2013.04.009 23602970 PMC3729797

[B72] Grigoriev I. Gouveia S. M. Van Der Vaart B. Demmers J. Smyth J. T. Honnappa S. (2008). STIM1 is a MT-plus-end-tracking protein involved in remodeling of the ER. *Curr. Biol.* 18 177–182. 10.1016/j.cub.2007.12.050 18249114 PMC2600655

[B73] Guimaraes S. C. Schuster M. Bielska E. Dagdas G. Kilaru S. Meadows B. R. (2015). Peroxisomes, lipid droplets, and endoplasmic reticulum “hitchhike” on motile early endosomes. *J. Cell. Biol.* 211 945–954. 10.1083/jcb.201505086 26620910 PMC4674278

[B74] Guo Y. Li D. Zhang S. Yang Y. Liu J. J. Wang X. (2018). Visualizing intracellular organelle and cytoskeletal interactions at nanoscale resolution on millisecond timescales. *Cell* 175 1430.e17–1442.e17. 30454650 10.1016/j.cell.2018.09.057

[B75] Gutierrez-Beltran E. Moschou P. N. Smertenko A. P. Bozhkov P. V. (2015). Tudor staphylococcal nuclease links formation of stress granules and processing bodies with mRNA catabolism in *Arabidopsis*. *Plant Cell* 27 926–943. 10.1105/tpc.114.134494 25736060 PMC4558657

[B76] Gutnick A. Banghart M. R. West E. R. Schwarz T. L. (2019). The light-sensitive dimerizer zapalog reveals distinct modes of immobilization for axonal mitochondria. *Nat. Cell. Biol.* 21 768–777. 10.1038/s41556-019-0317-2 31061466 PMC6662610

[B77] Hamada T. Tominaga M. Fukaya T. Nakamura M. Nakano A. Watanabe Y. (2012). Rna processing bodies, peroxisomes, Golgi bodies, mitochondria, and endoplasmic reticulum tubule junctions frequently pause at cortical microtubules. *Plant Cell Physiol.* 53 699–708. 10.1093/pcp/pcs025 22383625

[B78] Hamada T. Yako M. Minegishi M. Sato M. Kamei Y. Yanagawa Y. (2018). Stress granule formation is induced by a threshold temperature rather than a temperature difference in *Arabidopsis*. *J. Cell. Sci.* 131:jcs216051. 10.1242/jcs.216051 30030372

[B79] Hatch A. L. Gurel P. S. Higgs H. N. (2014). Novel roles for actin in mitochondrial fission. *J. Cell. Sci.* 127 4549–4560. 10.1242/jcs.153791 25217628 PMC4215709

[B80] Hernandez-Vega A. Braun M. Scharrel L. Jahnel M. Wegmann S. Hyman B. T. (2017). Local nucleation of microtubule bundles through tubulin concentration into a condensed tau phase. *Cell. Rep.* 20 2304–2312. 10.1016/j.celrep.2017.08.042 28877466 PMC5828996

[B81] Higuchi Y. Ashwin P. Roger Y. Steinberg G. (2014). Early endosome motility spatially organizes polysome distribution. *J. Cell. Biol.* 204 343–357. 10.1083/jcb.201307164 24493587 PMC3912533

[B82] Hirokawa N. Noda Y. Tanaka Y. Niwa S. (2009). Kinesin superfamily motor proteins and intracellular transport. *Nat. Rev. Mol. Cell. Biol.* 10 682–696. 10.1038/nrm2774 19773780

[B83] Holbro N. Grunditz A. Oertner T. G. (2009). Differential distribution of endoplasmic reticulum controls metabotropic signaling and plasticity at hippocampal synapses. *Proc. Natl. Acad. Sci. U.S.A.* 106 15055–15060. 10.1073/pnas.0905110106 19706463 PMC2736455

[B84] Holt C. E. Bullock S. L. (2009). Subcellular mRNA localization in animal cells and why it matters. *Science* 326 1212–1216. 10.1126/science.1176488 19965463 PMC3785123

[B85] Horvathova I. Voigt F. Kotrys A. V. Zhan Y. Artus-Revel C. G. Eglinger J. (2017). The dynamics of mRNA turnover revealed by single-molecule imaging in single cells. *Mol. Cell.* 68 615.e9–625.e9. 29056324 10.1016/j.molcel.2017.09.030

[B86] Hu X. Viesselmann C. Nam S. Merriam E. Dent E. W. (2008). Activity-dependent dynamic microtubule invasion of dendritic spines. *J. Neurosci.* 28 13094–13105. 10.1523/jneurosci.3074-08.2008 19052200 PMC6671621

[B87] Huang L. Mollet S. Souquere S. Le Roy F. Ernoult-Lange M. Pierron G. (2011). Mitochondria associate with P-bodies and modulate MicroRNA-mediated RNA interference. *J. Biol. Chem.* 286 24219–24230. 10.1074/jbc.m111.240259 21576251 PMC3129203

[B88] Hubstenberger A. Courel M. Bénard M. Souquere S. Ernoult-Lange M. Chouaib R. (2017). P-body purification reveals the condensation of repressed mRNA regulons. *Mol. Cell.* 68 144.e5–157.e5. 28965817 10.1016/j.molcel.2017.09.003

[B89] Ivanov P. A. Chudinova E. M. Nadezhdina E. S. (2003). Disruption of microtubules inhibits cytoplasmic ribonucleoprotein stress granule formation. *Exp. Cell Res.* 290 227–233. 10.1016/s0014-4827(03)00290-8 14567982

[B90] Jankowski S. Pohlmann T. Baumann S. Muntjes K. Devan S. K. Zander S. (2019). The multi Pam2 protein Upa2 functions as novel core component of endosomal mRNA transport. *EMBO Rep.* 20:e47381. 10.15252/embr.201847381 PMC6726905 31338952

[B91] Jaworski J. Kapitein L. C. Gouveia S. M. Dortland B. R. Wulf P. S. Grigoriev I. (2009). Dynamic microtubules regulate dendritic spine morphology and synaptic plasticity. *Neuron* 61 85–100. 10.1016/j.neuron.2008.11.013 19146815

[B92] Jeffery W. R. Tomlinson C. R. Brodeur R. D. (1983). Localization of actin messenger RNA during early ascidian development. *Dev. Biol.* 99 408–417. 10.1016/0012-1606(83)90290-7 6194032

[B93] Jiang H. Wang S. Huang Y. He X. Cui H. Zhu X. (2015). Phase transition of spindle-associated protein regulate spindle apparatus assembly. *Cell* 163 108–122. 10.1016/j.cell.2015.08.010 26388440 PMC4607269

[B94] Joensuu M. Belevich I. Ramo O. Nevzorov I. Vihinen H. Puhka M. (2014). Er sheet persistence is coupled to myosin 1c-regulated dynamic actin filament arrays. *Mol. Biol. Cell.* 25 1111–1126. 10.1091/mbc.e13-12-0712 24523293 PMC3967974

[B95] Kanai Y. Dohmae N. Hirokawa N. (2004). Kinesin transports RNA: isolation and characterization of an Rna-transporting granule. *Neuron* 43 513–525. 10.1016/j.neuron.2004.07.022 15312650

[B96] Kedersha N. Stoecklin G. Ayodele M. Yacono P. Lykke-Andersen J. Fritzler M. J. (2005). Stress granules and processing bodies are dynamically linked sites of mRNP remodeling. *J. Cell Biol.* 169 871–884. 10.1083/jcb.200502088 15967811 PMC2171635

[B97] Khong A. Matheny T. Jain S. Mitchell S. F. Wheeler J. R. Parker R. (2017). The stress granule transcriptome reveals principles of mRNA accumulation in stress granules. *Mol. Cell.* 68 808.e5–820.e5. 29129640 10.1016/j.molcel.2017.10.015 PMC5728175

[B98] Kilchert C. Weidner J. Prescianotto-Baschong C. Spang A. (2010). Defects in the secretory pathway and high Ca2+ induce multiple P-bodies. *Mol. Biol. Cell.* 21 2624–2638. 10.1091/mbc.e10-02-0099 20519435 PMC2912349

[B99] Kim S. Kalappurakkal J. M. Mayor S. Rosen M. K. (2019). Phosphorylation of nephrin induces phase separated domains that move through actomyosin contraction. *Mol. Biol. Cell.* 30 2996–3012. 10.1091/mbc.e18-12-0823 31599693 PMC6857567

[B100] King M. R. Petry S. (2020). Phase separation of TPX2 enhances and spatially coordinates microtubule nucleation. *Nat. Commun.* 11:270. 10.1038/s41467-019-14087-0 PMC6959270 31937751

[B101] Knowles R. B. Sabry J. H. Martone M. E. Deerinck T. J. Ellisman M. H. Bassell G. J. (1996). Translocation of RNA granules in living neurons. *J. Neurosci.* 16 7812–7820. 10.1523/jneurosci.16-24-07812.1996 8987809 PMC6579227

[B102] Kolobova E. Efimov A. Kaverina I. Rishi A. K. Schrader J. W. Ham A. J. (2009). Microtubule-dependent association of AKAP350A and CCAR1 with RNA stress granules. *Exp. Cell Res.* 315 542–555. 10.1016/j.yexcr.2008.11.011 19073175 PMC2788823

[B103] Konietzny A. Grendel J. Hertrich N. Dekkers D. H. W. Demmers J. A. A. Mikhaylova M. (2020). Synaptic anchoring of the endoplasmic reticulum depends on myosin V and caldendrin activity. *bioRxiv* [Preprint]. 10.1101/2020.08.14.250746

[B104] Konig J. Baumann S. Koepke J. Pohlmann T. Zarnack K. Feldbrugge M. (2009). The fungal RNA-binding protein Rrm4 mediates long-distance transport of ubi1 and rho3 mRNAs. *EMBO J.* 28 1855–1866. 10.1038/emboj.2009.145 19494833 PMC2711182

[B105] Korobova F. Ramabhadran V. Higgs H. N. (2013). An actin-dependent step in mitochondrial fission mediated by the ER-associated formin INF2. *Science* 339 464–467. 10.1126/science.1228360 23349293 PMC3843506

[B106] Kuijpers M. Hoogenraad C. C. (2011). Centrosomes, microtubules and neuronal development. *Mol. Cell. Neurosci.* 48 349–358. 10.1016/j.mcn.2011.05.004 21722732

[B107] Kulic I. M. Brown A. E. Kim H. Kural C. Blehm B. Selvin P. R. (2008). The role of microtubule movement in bidirectional organelle transport. *Proc. Natl. Acad. Sci. U.S.A.* 105 10011–10016. 10.1073/pnas.0800031105 18626022 PMC2481308

[B108] Kwon S. Zhang Y. Matthias P. (2007). The deacetylase HDAC6 is a novel critical component of stress granules involved in the stress response. *Genes Dev.* 21 3381–3394. 10.1101/gad.461107 18079183 PMC2113037

[B109] Lam S. S. Martell J. D. Kamer K. J. Deerinck T. J. Ellisman M. H. Mootha V. K. (2015). Directed evolution of APEX2 for electron microscopy and proximity labeling. *Nat. Methods* 12 51–54. 10.1038/nmeth.3179 25419960 PMC4296904

[B110] Lee J. E. Cathey P. I. Wu H. Parker R. Voeltz G. K. (2020). Endoplasmic reticulum contact sites regulate the dynamics of membraneless organelles. *Science* 367:eaay7108. 10.1126/science.aay7108 32001628 PMC10088059

[B111] Lee J. E. Westrate L. M. Wu H. Page C. Voeltz G. K. (2016). Multiple dynamin family members collaborate to drive mitochondrial division. *Nature* 540 139–143. 10.1038/nature20555 27798601 PMC5656044

[B112] Lee Y. S. Pressman S. Andress A. P. Kim K. White J. L. Cassidy J. J. (2009). Silencing by small RNAs is linked to endosomal trafficking. *Nat. Cell. Biol.* 11 1150–1156. 10.1038/ncb1930 19684574 PMC2737091

[B113] Liao Y. C. Fernandopulle M. S. Wang G. Choi H. Hao L. Drerup C. M. (2019). RNA granules hitchhike on lysosomes for long-distance transport, using annexin A11 as a molecular tether. *Cell* 179 147.e20–164.e20. 31539493 10.1016/j.cell.2019.08.050 PMC6890474

[B114] Lifland A. W. Zurla C. Yu J. Santangelo P. J. (2011). Dynamics of native beta-actin mRNA transport in the cytoplasm. *Traffic* 12 1000–1011. 10.1111/j.1600-0854.2011.01209.x 21518164 PMC3134163

[B115] Lin Y. Mori E. Kato M. Xiang S. Wu L. Kwon I. (2016). Toxic PR poly-dipeptides encoded by the C9orf72 repeat expansion target LC domain polymers. *Cell* 167 789.e12–802.e12. 27768897 10.1016/j.cell.2016.10.003 PMC5076566

[B116] Lindsay A. J. McCaffrey M. W. (2011). Myosin Va is required for P body but not stress granule formation. *J. Biol. Chem.* 286 11519–11528. 10.1074/jbc.m110.182808 21245139 PMC3064206

[B117] Litman P. Barg J. Ginzburg I. (1994). Microtubules are involved in the localization of tau mRNA in primary neuronal cell cultures. *Neuron* 13 1463–1474. 10.1016/0896-6273(94)90432-4 7993638

[B118] Liu X. Guo X. Niu L. Li X. Sun F. Hu J. (2019). Atlastin-1 regulates morphology and function of endoplasmic reticulum in dendrites. *Nat. Commun.* 10:568. 10.1038/s41467-019-08478-6 PMC6362286 30718476

[B119] Long R. M. Singer R. H. Meng X. Gonzalez I. Nasmyth K. Jansen R. P. (1997). Mating type switching in yeast controlled by asymmetric localization of ASH1 mRNA. *Science* 277 383–387. 10.1126/science.277.5324.383 9219698

[B120] Loschi M. Leishman C. C. Berardone N. Boccaccio G. L. (2009). Dynein and kinesin regulate stress-granule and P-body dynamics. *J. Cell. Sci.* 122 3973–3982. 10.1242/jcs.051383 19825938 PMC2773196

[B121] Lowery J. Jain N. Kuczmarski E. R. Mahammad S. Goldman A. Gelfand V. I. (2016). Abnormal intermediate filament organization alters mitochondrial motility in giant axonal neuropathy fibroblasts. *Mol. Biol. Cell.* 27 608–616. 10.1091/mbc.e15-09-0627 26700320 PMC4750921

[B122] Lu M. Van Tartwijk F. W. Lin J. Q. Nijenhuis W. Parutto P. Fantham M. (2020). The structure and global distribution of the endoplasmic reticulum network is actively regulated by lysosomes. *BioRxiv* [Preprint]. 10.1101/2020.01.15.907444 PMC7744115 33328230

[B123] Luo Y. Na Z. Slavoff S. A. (2018). P-bodies: composition. *Propert. Funct. Biochem.* 57 2424–2431. 10.1021/acs.biochem.7b01162 PMC6296482 29381060

[B124] Ma W. Mayr C. (2018). A membraneless organelle associated with the endoplasmic reticulum enables 3’utr-mediated protein-protein interactions. *Cell* 175 1492.e19–1506.e19. 30449617 10.1016/j.cell.2018.10.007 PMC6711188

[B125] Ma W. Zhen G. Xie W. Mayr C. (2020). Unstructured mRNAs form multivalent RNA-RNA interactions to generate TIS granule networks. *bioRxiv* [Preprint]. 10.1101/2020.02.14.949503v1

[B126] Man Y. Kanso E. (2019). Morphological transitions of axially-driven microfilaments. *Soft. Matter.* 15 5163–5173. 10.1039/c8sm02397b 31215548

[B127] Markmiller S. Soltanieh S. Server K. L. Mak R. Jin W. Fang M. Y. (2018). Context-dependent and disease-specific diversity in protein interactions within stress granules. *Cell* 172 590.e13–604.e13. 29373831 10.1016/j.cell.2017.12.032 PMC5969999

[B128] Martin-Cofreces N. B. Sanchez-Madrid F. (2018). Sailing to and docking at the immune synapse: role of tubulin dynamics and molecular motors. *Front. Immunol.* 9:1174. 10.3389/fimmu.2018.01174 29910809 PMC5992405

[B129] Mateju D. Eichenberger B. Eglinger J. Roth G. Chao J. A. (2020). Single-molecule imaging reveals translation of mrnas localized to stress granules. *bioRxiv* [Preprint]. 10.1101/2020.03.31.018093v1 33308477

[B130] Matheny T. Rao B. S. Parker R. (2019). Transcriptome-wide comparison of stress granules and P-bodies reveals that translation plays a major role in RNA partitioning. *Mol. Cell. Biol.* 39:e00313-19. 10.1128/MCB.00313-19 PMC6879202 31591142

[B131] Matveeva E. A. Venkova L. S. Chernoivanenko I. S. Minin A. A. (2015). Vimentin is involved in regulation of mitochondrial motility and membrane potential by Rac1. *Biol. Open* 4 1290–1297. 10.1242/bio.011874 26369929 PMC4610213

[B132] McClintock M. A. Dix C. I. Johnson C. M. Mclaughlin S. H. Maizels R. J. Hoang H. T. (2018). RNA-directed activation of cytoplasmic dynein-1 in reconstituted transport RNPs. *eLife* 7:e36312. 10.7554/eLife.36312 PMC6056234 29944118

[B133] Mehta K. Chacko L. A. Chug M. K. Jhunjhunwala S. Ananthanarayanan V. (2019). Association of mitochondria with microtubules inhibits mitochondrial fission by precluding assembly of the fission protein Dnm1. *J. Biol. Chem.* 294 3385–3396. 10.1074/jbc.ra118.006799 30602572 PMC6416417

[B134] Melemedjian O. K. Mejia G. L. Lepow T. S. Zoph O. K. Price T. J. (2014). Bidirectional regulation of P body formation mediated by eIF4F complex formation in sensory neurons. *Neurosci. Lett.* 563 169–174. 10.1016/j.neulet.2013.09.048 24080374 PMC3951963

[B135] Messitt T. J. Gagnon J. A. Kreiling J. A. Pratt C. A. Yoon Y. J. Mowry K. L. (2008). Multiple kinesin motors coordinate cytoplasmic RNA transport on a subpopulation of microtubules in *Xenopus oocytes*. *Dev. Cell.* 15 426–436. 10.1016/j.devcel.2008.06.014 18771961 PMC2581415

[B136] Mofatteh M. Bullock S. L. (2017). SnapShot: subcellular mRNA localization. *Cell* 169 178.e–178.e. 28340345 10.1016/j.cell.2017.03.004

[B137] Mohan R. John A. (2015). Microtubule-associated proteins as direct crosslinkers of actin filaments and microtubules. *IUBMB Life* 67 395–403. 10.1002/iub.1384 26104829

[B138] Montenegro Gouveia S. Zitouni S. Kong D. Duarte P. Ferreira Gomes B. Sousa A. L. (2018). Plk4 is a microtubule-associated protein that self-assembles promoting de novo MTOC formation. *J. Cell Sci.* 132:jcs219501. 10.1242/jcs.219501 30237222 PMC6398482

[B139] Moon S. L. Morisaki T. Khong A. Lyon K. Parker R. Stasevich T. J. (2019). Multicolour single-molecule tracking of mRNA interactions with RNP granules. *Nat. Cell. Biol.* 21 162–168. 10.1038/s41556-018-0263-4 30664789 PMC6375083

[B140] Nadezhdina E. S. Lomakin A. J. Shpilman A. A. Chudinova E. M. Ivanov P. A. (2010). Microtubules govern stress granule mobility and dynamics. *Biochim. Biophys. Acta* 1803 361–371. 10.1016/j.bbamcr.2009.12.004 20036288

[B141] Nguyen M. M. Mccracken C. J. Milner E. S. Goetschius D. J. Weiner A. T. Long M. K. (2014). Gamma-tubulin controls neuronal microtubule polarity independently of Golgi outposts. *Mol. Biol. Cell.* 25 2039–2050. 10.1091/mbc.e13-09-0515 24807906 PMC4072577

[B142] Nixon-Abell J. Obara C. J. Weigel A. V. Li D. Legant W. R. Xu C. S. (2016). Increased spatiotemporal resolution reveals highly dynamic dense tubular matrices in the peripheral ER. *Science* 354:aaf3928. 10.1126/science.aaf3928 27789813 PMC6528812

[B143] Nunnari J. Suomalainen A. (2012). Mitochondria: in sickness and in health. *Cell* 148 1145–1159. 10.1016/j.cell.2012.02.035 22424226 PMC5381524

[B144] Oddoux S. Zaal K. J. Tate V. Kenea A. Nandkeolyar S. A. Reid E. (2013). Microtubules that form the stationary lattice of muscle fibers are dynamic and nucleated at Golgi elements. *J. Cell. Biol.* 203 205–213. 10.1083/jcb.201304063 24145165 PMC3812964

[B145] Oh J. Y. Kwon A. Jo A. Kim H. Goo Y. S. Lee J. A. (2013). Activity-dependent synaptic localization of processing bodies and their role in dendritic structural plasticity. *J. Cell. Sci.* 126 2114–2123. 10.1242/jcs.125690 23487039

[B146] Olgeiser L. Haag C. Boerner S. Ule J. Busch A. Koepke J. (2019). The key protein of endosomal mrnp transport Rrm4 binds translational landmark sites of cargo mRNAs. *EMBO Rep.* 20:e46588. 10.15252/embr.201846588 PMC6322384 30552148

[B147] Ong J. Y. Torres J. Z. (2020). Phase separation in cell division. *Mol. Cell.* 80 9–20. 10.1016/j.molcel.2020.08.007 32860741 PMC7541545

[B148] Ori-McKenney K. M. Jan L. Y. Jan Y. N. (2012). Golgi outposts shape dendrite morphology by functioning as sites of acentrosomal microtubule nucleation in neurons. *Neuron* 76 921–930. 10.1016/j.neuron.2012.10.008 23217741 PMC3523279

[B149] Ornelles D. A. Fey E. G. Penman S. (1986). Cytochalasin releases mRNA from the cytoskeletal framework and inhibits protein synthesis. *Mol. Cell. Biol.* 6 1650–1662. 10.1128/mcb.6.5.1650 3785175 PMC367692

[B150] Özkan N. Koppers M. Van Soest I. Van Harten A. Liv N. Klumperman J. (2020). ER – lysosome contacts at a pre-axonal region regulate axonal lysosome availability. *bioRxiv* [Preprint]. 10.1101/2020.06.16.153734 PMC8302662 34301956

[B151] Park S. H. Zhu P. P. Parker R. L. Blackstone C. (2010). Hereditary spastic paraplegia proteins Reep1, spastin, and atlastin-1 coordinate microtubule interactions with the tubular ER network. *J. Clin. Invest.* 120 1097–1110. 10.1172/jci40979 20200447 PMC2846052

[B152] Patel P. H. Barbee S. A. Blankenship J. T. (2016). GW-bodies and P-bodies constitute two separate pools of sequestered non-translating RNAS. *PLoS One* 11:e0150291. 10.1371/journal.pone.0150291 26930655 PMC4773245

[B153] Pattabiraman S. Azad G. K. Amen T. Brielle S. Park J. E. Sze S. K. (2020). Vimentin protects differentiating stem cells from stress. *Sci. Rep.* 10:19525. 10.1038/s41598-020-76076-4 PMC7658978 33177544

[B154] Pavez M. Thompson A. C. Arnott H. J. Mitchell C. B. D’atri I. Don E. K. (2019). STIM1 is required for remodeling of the endoplasmic reticulum and microtubule cytoskeleton in steering growth cones. *J. Neurosci.* 39 5095–5114. 10.1523/jneurosci.2496-18.2019 31023836 PMC6595949

[B155] Pchitskaya E. Kraskovskaya N. Chernyuk D. Popugaeva E. Zhang H. Vlasova O. (2017). Stim2-Eb3 association and morphology of dendritic spines in hippocampal neurons. *Sci. Rep.* 7:17625. 10.1038/s41598-017-17762-8 PMC5732248 29247211

[B156] Pease-Raissi S. E. Pazyra-Murphy M. F. Li Y. Wachter F. Fukuda Y. Fenstermacher S. J. (2017). Paclitaxel reduces axonal Bclw to initiate Ip3R1-dependent axon degeneration. *Neuron* 96 373.e6–386.e6. 29024661 10.1016/j.neuron.2017.09.034 PMC5680044

[B157] Perez-Alvarez A. Yin S. Schulze C. Hammer J. A. Wagner W. Oertner T. G. (2020). Endoplasmic reticulum visits highly active spines and prevents runaway potentiation of synapses. *Nat. Commun.* 11:5083. 10.1038/s41467-020-18889-5 PMC7546627 33033259

[B158] Phillips M. J. Voeltz G. K. (2016). Structure and function of ER membrane contact sites with other organelles. *Nat. Rev. Mol. Cell. Biol.* 17 69–82. 10.1038/nrm.2015.8 26627931 PMC5117888

[B159] Pilling A. D. Horiuchi D. Lively C. M. Saxton W. M. (2006). Kinesin-1 and Dynein are the primary motors for fast transport of mitochondria in Drosophila motor axons. *Mol. Biol. Cell.* 17 2057–2068. 10.1091/mbc.e05-06-0526 16467387 PMC1415296

[B160] Pohlmann T. Baumann S. Haag C. Albrecht M. Feldbrugge M. (2015). A FYVE zinc finger domain protein specifically links mrna transport to endosome trafficking. *eLife* 4:e06041. 10.7554/eLife.06041 PMC4466420 25985087

[B161] Popovic D. Nijenhuis W. Kapitein L. C. Pelkmans L. (2020). Co-translational targeting of transcripts to endosomes. *bioRxiv* [Preprint].

[B162] Preitner N. Quan J. Nowakowski D. W. Hancock M. L. Shi J. Tcherkezian J. (2014). APC is an RNA-binding protein, and its interactome provides a link to neural development and microtubule assembly. *Cell* 158 368–382. 10.1016/j.cell.2014.05.042 25036633 PMC4133101

[B163] Raiborg C. Wenzel E. M. Pedersen N. M. Olsvik H. Schink K. O. Schultz S. W. (2015). Repeated ER-endosome contacts promote endosome translocation and neurite outgrowth. *Nature* 520 234–238. 10.1038/nature14359 25855459

[B164] Rajgor D. Mellad J. A. Soong D. Rattner J. B. Fritzler M. J. Shanahan C. M. (2014). Mammalian microtubule P-body dynamics are mediated by nesprin-1. *J. Cell. Biol.* 205 457–475. 10.1083/jcb.201306076 24862572 PMC4033771

[B165] Rapoport T. A. (2007). Protein translocation across the eukaryotic endoplasmic reticulum and bacterial plasma membranes. *Nature* 450 663–669. 10.1038/nature06384 18046402

[B166] Riggs C. L. Kedersha N. Ivanov P. Anderson P. (2020). Mammalian stress granules and P bodies at a glance. *J. Cell Sci.* 133:jcs242487. 10.1242/jcs.242487 32873715 PMC10679417

[B167] Rodriguez O. C. Schaefer A. W. Mandato C. A. Forscher P. Bement W. M. Waterman-Storer C. M. (2003). Conserved microtubule-actin interactions in cell movement and morphogenesis. *Nat. Cell. Biol.* 5 599–609. 10.1038/ncb0703-599 12833063

[B168] Rodriguez-Garcia R. Volkov V. A. Chen C. Y. Katrukha E. A. Olieric N. Aher A. (2020). Mechanisms of motor-independent membrane remodeling driven by dynamic microtubules. *Curr. Biol.* 30 972.e12–987.e12. 32032506 10.1016/j.cub.2020.01.036 PMC7090928

[B169] Rogers S. L. Gelfand V. I. (2000). Membrane trafficking, organelle transport, and the cytoskeleton. *Curr. Opin. Cell. Biol.* 12 57–62. 10.1016/s0955-0674(99)00057-5 10679352

[B170] Rowland A. A. Chitwood P. J. Phillips M. J. Voeltz G. K. (2014). ER contact sites define the position and timing of endosome fission. *Cell* 159 1027–1041. 10.1016/j.cell.2014.10.023 25416943 PMC4634643

[B171] Sabari B. R. Dall’agnese A. Young R. A. (2020). Biomolecular condensates in the nucleus. *Trends Biochem. Sci.* 45 961–977. 10.1016/j.tibs.2020.06.007 32684431 PMC7572565

[B172] Safari M. S. King M. R. Brangwynne C. P. Petry S. (2020). Branching microtubule nucleation is controlled by importin-mediated inhibition of TPX2 phase separation. *bioRxiv* [Preprint]. 10.1101/2020.09.01.276469

[B173] Sahoo P. K. Lee S. J. Jaiswal P. B. Alber S. Kar A. N. Miller-Randolph S. (2018). Axonal G3BP1 stress granule protein limits axonal mrna translation and nerve regeneration. *Nat. Commun.* 9:3358. 10.1038/s41467-018-05647-x PMC6105716 30135423

[B174] Salogiannis J. Reck-Peterson S. L. (2017). Hitchhiking: a non-canonical mode of microtubule-based transport. *Trends Cell. Biol.* 27 141–150. 10.1016/j.tcb.2016.09.005 27665063 PMC5258766

[B175] Schmid M. Jaedicke A. Du T. G. Jansen R. P. (2006). Coordination of endoplasmic reticulum and mrna localization to the yeast bud. *Curr. Biol.* 16 1538–1543. 10.1016/j.cub.2006.06.025 16890529

[B176] Schwarz D. S. Blower M. D. (2016). The endoplasmic reticulum: structure, function and response to cellular signaling. *Cell Mol. Life Sci.* 73 79–94. 10.1007/s00018-015-2052-6 26433683 PMC4700099

[B177] Scorrano L. De Matteis M. A. Emr S. Giordano F. Hajnoczky G. Kornmann B. (2019). Coming together to define membrane contact sites. *Nat. Commun.* 10:1287. 10.1038/s41467-019-09253-3 PMC6427007 30894536

[B178] Sehgal P. B. Westley J. Lerea K. M. Disenso-Browne S. Etlinger J. D. (2020). Biomolecular condensates in cell biology and virology: phase-separated membraneless organelles (MLOS). *Anal. Biochem.* 597:113691. 10.1016/j.ab.2020.113691 32194074

[B179] Sheth U. Parker R. (2003). Decapping and decay of messenger RNA occur in cytoplasmic processing bodies. *Science* 300 805–808. 10.1126/science.1082320 12730603 PMC1876714

[B180] Shibata Y. Voeltz G. K. Rapoport T. A. (2006). Rough sheets and smooth tubules. *Cell* 126 435–439. 10.1016/j.cell.2006.07.019 16901774

[B181] Sleigh J. N. Rossor A. M. Fellows A. D. Tosolini A. P. Schiavo G. (2019). Axonal transport and neurological disease. *Nat. Rev. Neurol.* 15 691–703. 31558780 10.1038/s41582-019-0257-2

[B182] Smirnova E. Griparic L. Shurland D. L. van der Bliek A. M. (2001). Dynamin-related protein Drp1 is required for mitochondrial division in mammalian cells. *Mol. Biol. Cell.* 12 2245–2256. 10.1091/mbc.12.8.2245 11514614 PMC58592

[B183] Smolina N. Khudiakov A. Knyazeva A. Zlotina A. Sukhareva K. Kondratov K. (2020). Desmin mutations result in mitochondrial dysfunction regardless of their aggregation properties. *Biochim. Biophys. Acta Mol. Basis Dis.* 1866:165745. 10.1016/j.bbadis.2020.165745 32105824

[B184] So C. Seres K. B. Steyer A. M. Monnich E. Clift D. Pejkovska A. (2019). A liquid-like spindle domain promotes acentrosomal spindle assembly in mammalian oocytes. *Science* 364:eaat9557. 10.1126/science.aat9557 31249032 PMC6629549

[B185] Steffen J. Koehler C. M. (2018). ER-mitochondria contacts: actin dynamics at the ER control mitochondrial fission via calcium release. *J. Cell. Biol.* 217 15–17. 10.1083/jcb.201711075 29259094 PMC5748997

[B186] Steffens A. Jaegle B. Tresch A. Hulskamp M. Jakoby M. (2014). Processing-body movement in *Arabidopsis* depends on an interaction between myosins and Decapping Protein1. *Plant. Physiol.* 164 1879–1892. 10.1104/pp.113.233031 24525673 PMC3982750

[B187] Su X. Ditlev J. A. Hui E. Xing W. Banjade S. Okrut J. (2016). Phase separation of signaling molecules promotes T cell receptor signal transduction. *Science* 352 595–599. 10.1126/science.aad9964 27056844 PMC4892427

[B188] Sweet T. J. Boyer B. Hu W. Baker K. E. Coller J. (2007). Microtubule disruption stimulates P-body formation. *RNA* 13 493–502. 10.1261/rna.355807 17307817 PMC1831866

[B189] Szaflarski W. Fay M. M. Kedersha N. Zabel M. Anderson P. Ivanov P. (2016). Vinca alkaloid drugs promote stress-induced translational repression and stress granule formation. *Oncotarget* 7 30307–30322. 10.18632/oncotarget.8728 27083003 PMC5058682

[B190] Takagishi Y. Oda S. Hayasaka S. Dekker-Ohno K. Shikata T. Inouye M. (1996). The dilute-lethal (dl) gene attacks a Ca2+ store in the dendritic spine of Purkinje cells in mice. *Neurosci. Lett.* 215 169–172. 10.1016/0304-3940(96)12967-0 8899740

[B191] Takizawa P. A. Sil A. Swedlow J. R. Herskowitz I. Vale R. D. (1997). Actin-dependent localization of an RNA encoding a cell-fate determinant in yeast. *Nature* 389 90–93. 10.1038/38015 9288973

[B192] Tauber D. Tauber G. Parker R. (2020). Mechanisms and regulation of RNA condensation in RNP granule formation. *Trends Biochem. Sci.* 45 764–778. 10.1016/j.tibs.2020.05.002 32475683 PMC7211619

[B193] Teixeira D. Sheth U. Valencia-Sanchez M. A. Brengues M. Parker R. (2005). Processing bodies require RNA for assembly and contain nontranslating mRNAs. *RNA* 11 371–382. 10.1261/rna.7258505 15703442 PMC1370727

[B194] Terasaki M. Chen L. B. Fujiwara K. (1986). Microtubules and the endoplasmic reticulum are highly interdependent structures. *J. Cell. Biol.* 103 1557–1568. 10.1083/jcb.103.4.1557 3533956 PMC2114338

[B195] Toresson H. Grant S. G. (2005). Dynamic distribution of endoplasmic reticulum in hippocampal neuron dendritic spines. *Eur. J. Neurosci.* 22 1793–1798. 10.1111/j.1460-9568.2005.04342.x 16197520

[B196] Trcek T. Lehmann R. (2019). Germ granules in *Drosophila*. *Traffic* 20 650–660. 10.1111/tra.12674 31218815 PMC6771631

[B197] Tsai N. P. Tsui Y. C. Wei L. N. (2009). Dynein motor contributes to stress granule dynamics in primary neurons. *Neuroscience* 159 647–656. 10.1016/j.neuroscience.2008.12.053 19171178 PMC2650738

[B198] Valm A. M. Cohen S. Legant W. R. Melunis J. Hershberg U. Wait E. (2017). Applying systems-level spectral imaging and analysis to reveal the organelle interactome. *Nature* 546 162–167. 10.1038/nature22369 28538724 PMC5536967

[B199] van de Willige D. Hummel J. J. Alkemade C. Kahn O. I. Au F. K. Qi R. Z. (2019). Cytolinker Gas2L1 regulates axon morphology through microtubule-modulated actin stabilization. *EMBO Rep.* 20:e47732. 10.15252/embr.201947732 PMC6831992 31486213

[B200] van Spronsen M. Mikhaylova M. Lipka J. Schlager M. A. van den Heuvel D. J. Kuijpers M. (2013). TRAK/Milton motor-adaptor proteins steer mitochondrial trafficking to axons and dendrites. *Neuron* 77 485–502. 10.1016/j.neuron.2012.11.027 23395375

[B201] Vidaki M. Drees F. Saxena T. Lanslots E. Taliaferro M. J. Tatarakis A. (2017). A requirement for mena, an actin regulator, in local mRNA translation in developing neurons. *Neuron* 95 608.e5–622.e5. 28735747 10.1016/j.neuron.2017.06.048 PMC5616167

[B202] Voronina E. Seydoux G. Sassone-Corsi P. Nagamori I. (2011). RNA granules in germ cells. *Cold Spring Harb. Perspect. Biol.* 3:a002774. 10.1101/cshperspect.a002774 21768607 PMC3225947

[B203] Wagner W. Brenowitz S. D. Hammer J. A. III (2011). Myosin-Va transports the endoplasmic reticulum into the dendritic spines of Purkinje neurons. *Nat. Cell. Biol.* 13 40–48. 10.1038/ncb2132 21151132 PMC3403743

[B204] Wang C. Schmich F. Srivatsa S. Weidner J. Beerenwinkel N. Spang A. (2018). Context-dependent deposition and regulation of mRNAs in P-bodies. *eLife* 7:e29815. 10.7554/eLife.29815 PMC5752201 29297464

[B205] Wang N. Rapoport T. A. (2019). Reconstituting the reticular ER network - mechanistic implications and open questions. *J. Cell. Sci.* 132:jcs227611. 10.1242/jcs.227611 30670475

[B206] Waterman-Storer C. M. Salmon E. D. (1998). Endoplasmic reticulum membrane tubules are distributed by microtubules in living cells using three distinct mechanisms. *Curr. Biol.* 8 798–806. 10.1016/s0960-9822(98)70321-5 9663388

[B207] Wegmann S. Eftekharzadeh B. Tepper K. Zoltowska K. M. Bennett R. E. Dujardin S. (2018). Tau protein liquid-liquid phase separation can initiate tau aggregation. *EMBO J.* 37:e98049. 10.15252/embj.201798049 PMC5881631 29472250

[B208] Weidner J. Wang C. Prescianotto-Baschong C. Estrada A. F. Spang A. (2014). The polysome-associated proteins Scp160 and Bfr1 prevent P body formation under normal growth conditions. *J. Cell. Sci.* 127 1992–2004. 10.1242/jcs.142083 24569876

[B209] Weiner A. T. Seebold D. Y. Torres-Gutierrez P. Folker C. Swope R. D. Kothe G. O. (2020). Endosomal Wnt signaling proteins control microtubule nucleation in dendrites. *PLoS Biol.* 18:e3000647. 10.1371/journal.pbio.3000647 32163403 PMC7067398

[B210] Weirich K. L. Banerjee S. Dasbiswas K. Witten T. A. Vaikuntanathan S. Gardel M. L. (2017). Liquid behavior of cross-linked actin bundles. *Proc. Natl. Acad. Sci. U.S.A.* 114 2131–2136. 10.1073/pnas.1616133114 28202730 PMC5338483

[B211] Wheeler J. R. Matheny T. Jain S. Abrisch R. Parker R. (2016). Distinct stages in stress granule assembly and disassembly. *eLife* 5:e18413. 10.7554/eLife.18413 PMC5014549 27602576

[B212] Woodruff J. B. Ferreira Gomes B. Widlund P. O. Mahamid J. Honigmann A. Hyman A. A. (2017). The centrosome is a selective condensate that nucleates microtubules by concentrating tubulin. *Cell* 169 1066.e10–1077.e10. 28575670 10.1016/j.cell.2017.05.028

[B213] Woods L. C. Berbusse G. W. Naylor K. (2016). Microtubules are essential for mitochondrial dynamics-fission, fusion, and motility-in *Dictyostelium discoideum*. *Front. Cell. Dev. Biol.* 4:19. 10.3389/fcell.2016.00019 27047941 PMC4801864

[B214] Wozniak M. J. Bola B. Brownhill K. Yang Y. C. Levakova V. Allan V. J. (2009). Role of kinesin-1 and cytoplasmic dynein in endoplasmic reticulum movement in Vero cells. *J. Cell. Sci.* 122 1979–1989. 10.1242/jcs.041962 19454478 PMC2723153

[B215] Wu H. Zhou J. Zhu T. Cohen I. Dictenberg J. (2020). A kinesin adapter directly mediates dendritic mrna localization during neural development in mice. *J. Biol. Chem.* 295 6605–6628. 10.1074/jbc.ra118.005616 32111743 PMC7212647

[B216] Wu M. Kalyanasundaram A. Zhu J. (2013). Structural and biomechanical basis of mitochondrial movement in eukaryotic cells. *Int. J. Nanomed.* 8 4033–4042. 10.2147/ijn.s52132 24187495 PMC3810443

[B217] Wu X. Cai Q. Feng Z. Zhang M. (2020). Liquid-liquid phase separation in neuronal development and synaptic signaling. *Dev. Cell.* 55 18–29. 10.1016/j.devcel.2020.06.012 32726576

[B218] Wu Y. Whiteus C. Xu C. S. Hayworth K. J. Weinberg R. J. Hess H. F. (2017). Contacts between the endoplasmic reticulum and other membranes in neurons. *Proc. Natl. Acad. Sci. U.S.A.* 114 E4859–E4867. 28559323 10.1073/pnas.1701078114 PMC5474793

[B219] Youn J. Y. Dunham W. H. Hong S. J. Knight J. D. R. Bashkurov M. Chen G. I. (2018). High-density proximity mapping reveals the subcellular organization of mRNA-associated granules and bodies. *Mol. Cell.* 69 517.e11–532.e11. 29395067 10.1016/j.molcel.2017.12.020

[B220] Youn J. Y. Dyakov B. J. A. Zhang J. Knight J. D. R. Vernon R. M. Forman-Kay J. D. (2019). Properties of stress granule and P-body proteomes. *Mol. Cell.* 76 286–294. 10.1016/j.molcel.2019.09.014 31626750

[B221] Zacharogianni M. Aguilera-Gomez A. Veenendaal T. Smout J. Rabouille C. (2014). A stress assembly that confers cell viability by preserving ERES components during amino-acid starvation. *eLife* 3:e04132 10.7554/eLife.04132 PMC4270098 25386913

[B222] Zbinden A. Perez-Berlanga M. De Rossi P. Polymenidou M. (2020). Phase separation and neurodegenerative diseases: a disturbance in the force. *Dev. Cell.* 55 45–68. 10.1016/j.devcel.2020.09.014 33049211

[B223] Zeitelhofer M. Karra D. Macchi P. Tolino M. Thomas S. Schwarz M. (2008). Dynamic interaction between P-bodies and transport ribonucleoprotein particles in dendrites of mature hippocampal neurons. *J. Neurosci.* 28 7555–7562. 10.1523/jneurosci.0104-08.2008 18650333 PMC6670838

[B224] Zhang H. Hu J. (2016). Shaping the endoplasmic reticulum into a social network. *Trends Cell. Biol.* 26 934–943. 10.1016/j.tcb.2016.06.002 27339937

[B225] Zhao Y. G. Zhang H. (2020). Phase separation in membrane biology: the interplay between membrane-bound organelles and membraneless condensates. *Dev. Cell.* 55 30–44. 10.1016/j.devcel.2020.06.033 32726575

[B226] Zhou W. Chang J. Wang X. Savelieff M. G. Zhao Y. Ke S. (2014). GM130 is required for compartmental organization of dendritic golgi outposts. *Curr. Biol.* 24 1227–1233. 10.1016/j.cub.2014.04.008 24835455 PMC4047983

